# Chemical Synthesis and Biological Activities of Novel Pleuromutilin Derivatives with Substituted Amino Moiety

**DOI:** 10.1371/journal.pone.0082595

**Published:** 2013-12-23

**Authors:** Ruofeng Shang, Shengyu Wang, Ximing Xu, Yunpeng Yi, Wenzhu Guo, YuLiu, Jianping Liang

**Affiliations:** 1 Key Laboratory of New Animal Drug Project of Gansu Province, Key Laboratory of Veterinary Pharmacceutical Development, Ministry of Agriculture, Lanzhou Institute of Animal Science and Veterinary Pharmaceutics Science, Chinese Academy of Agricultural Sciences, Lanzhou, Gansu, China; 2 University Hospital of Gansu Traditional Chinese Medicine, Lanzhou, Gansu, China; 3 Université Paris Diderot, Sorbonne Paris Cité, Unité de Biologie Fonctionnelle et Adaptative, CNRS, Paris, France; University of Iowa Carver College of Medicine, United States of America

## Abstract

Novel pleuromutilin derivatives designed based on the structure of valnemulin were synthesized and evaluated for their *in vitro* antibacterial activities. These pleuromutilin derivatives with substituted amino moiety exhibited excellent activities against methicillin-resistant *Staphylococcus aureus,* methicillin-resistant *Staphylococcus epidermidis, Escherichia coli,* and *Streptococcus agalactiae.* Compound **5b** showed the highest antibacterial activities and even exceeded tiamulin. Moreover, the docking experiments provided information about the binding model between the synthesized compounds and peptidyl transferase center (PTC) of 23S rRNA.

## Introduction

In the last three decades the abuse of antibiotics has made more pathogenic bacteria resistance to drugs, which leads to many available drugs reducing or losing curative effect [1]. Drug-resistance bacteria, especially the *Staphylococcus aureus*, *Staphylococcus pneumoniae*, *Mycobacterium tuberculosis*, etc. endanger human health and poses an economic problem seriously [2]. The rapid emergence of drug-resistant bacteria urges research workers to identify and develop new antibacterial agents with novel mechanisms of action against drug-resistant bacterial strains.

**Figure 1:**
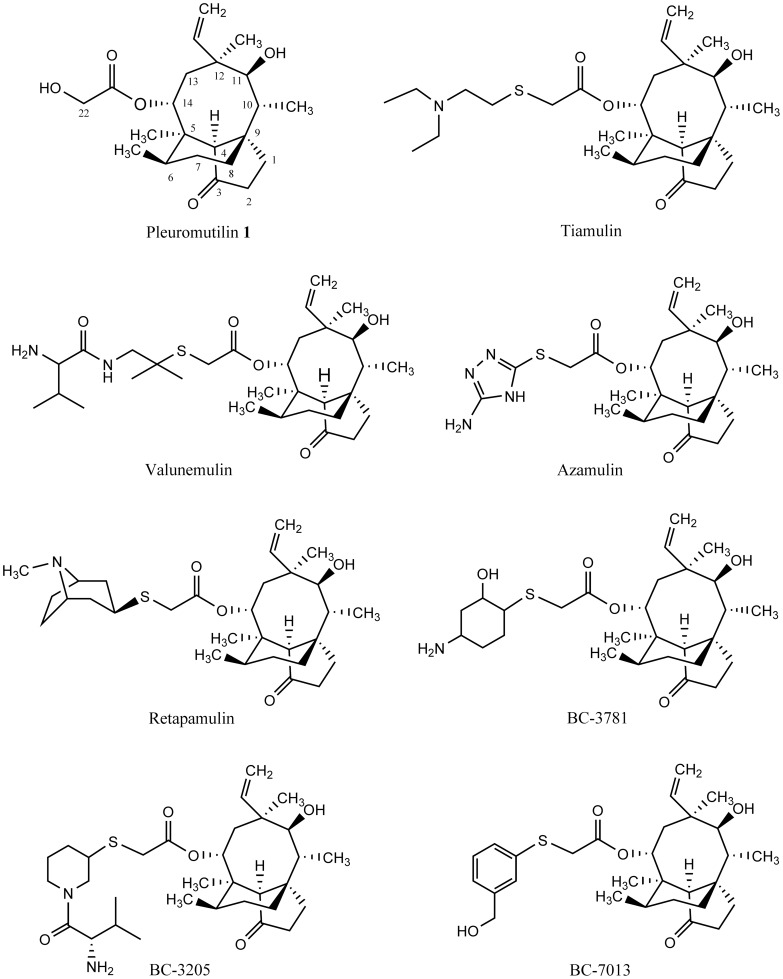
Structural formulas of pleuromutilin (1) and derivatives.

Pleuromutilin (**1**) (Figure 1) was first isolated in a crystalline form from cultures of two species of basidiomycetes, *Pleurotus mutilus* and *P. passeckerianus* in 1951 [3]. Pleuromutilin is a diterpene, constituted of a rather rigid 5–6–8 tricyclic carbon skeleton with eight stereogenic centers [4], [5]. Molecular modifications of the C-14 glycolic acid chain of pleuromutilin have led to two pleuromutilin derivatives, tiamulin and valnemulin (Figure 1) [6]. The two compounds have been successfully developed as therapeutic agents for veterinary use [7], [8]. During the early 1980s, extensive effort was made to formulate azamulin (Figure 1) for human use. Although azamulin showed good activity *in vitro* against many clinical isolates, it did not go into the stage for further clinic trial because of strongly inhibition of cytochrome P450 and terrible solubility in water [9], [10]. Retapamulin (Figure 1) became the first pleuromutilin approved for human use in 2007 by Food and Drug Administration (FDA) [11], [12]. Besides retapamulin, BC-3781, BC-3205 and BC-7013 (Figure 1) are developing for human use [13], [14].

Further studies have shown that pleuromutilin derivatives interfered with bacterial protein synthesis *via* a specific interaction with the 23S rRNA of the 50S bacterial ribosome subunit [15], [16]. The domain V of 23S rRNA at the peptidyl transferase center (PTC) is mutilins derivatives binding site, in which the tricyclic core of the pleuromutilin is positioned in a pocket close to the A-tRNA binding site, whereas the C-14 extension points toward the P-tRNA binding site [17]. Thus these compounds prevent the correct positioning of the tRNAs for peptide transfer, and inhibit the peptidyl transferase [6], [18].

Structure activity relationship (SAR) studies show that the presence of thioether group at C-22 position of pleuromutilin enhances antibacterial activity [19], [20]. The thioether group moiety is key to their pharmacological properties, especially with side chain [7], [17]. For example, antibacterial activity of valnemulin containing dimethyl propane moiety is more effective than that of tiamulin in vitro as well in vivo [21], [22]. Previous work in our group has led to the synthesis and analysis of antibacterial activity of 17 semisynthetic pleuromutilin derivatives bearing dimethyl propane moiety [23]. Based on the bioactivity studies it was proposed that the antibacterial activity of these compounds is connected with the alkaline group at the end of side chain.

As a part of our research work on the development of useful synthetic molecules, we have planned to introduce tertiary amine at the end of dimethyl propane moiety attached to the side chain at C-14 of pleuromutilin. Thus, the present study reports the synthesis, antibacterial studies, molecular docking of the synthesized compounds into 50S ribosomal subunit (PDB ID: 1XBP). In addition, we report here the single crystal X-ray study of **4** to understand its conformational feature and supramolecular assembly. It helps in understanding the exact 3D conformation of the molecule which would help in further studying the mechanism of action of the drug and also in docking studies with receptor.

## Results and Discussion

### Synthesis

**Figure 2:**
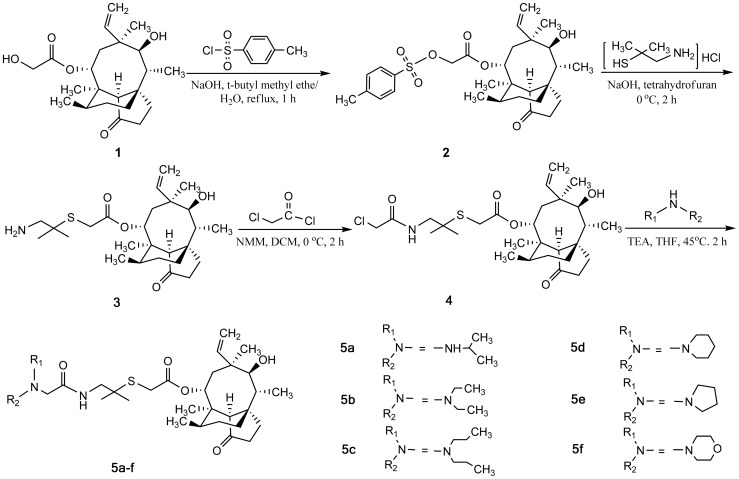
Scheme for the synthesis of target compounds 5a–f.

The reaction pathways used to synthesize the designed compounds (**5a–f**) were described in Figure 2. The pleuromutilin **1** was converted into the known *p*-toluenesulfonyl ester **2**, which by a nucleophilic substitution was further converted into an intermediate, 14-O-[(1-amino-2-methylpropane-2-yl) thioacetyl] mutilin **3**, in the manner previously reported by us [23]. The key intermediate, 14-O-[(2-chloroacetamide -2-methylpropane-2-yl) thioacetyl] mutilin **4**, was prepared by commercial available chloracetyl chloride and intermediate **3** with an aim to construct acetamide linker between the tertiary amine and 2-methylpropane. Then, intermediate **4** reacted with a series of secondary amines by the nucleophilic reaction in the presence of triethylamine to afford the corresponding target compounds **5a–f**. All the formed tertiary amines were treated by distilled water and saturated NaHCO_3_ washing, followed by purification with column chromatography and characterized by means of IR, ^1^H NMR, ^13^C NMR and HRMS spectral analysis (Details are provided in Figure S1).

### Biological Evaluation

The synthesized pleuromutilin derivatives **5a–f** were tested for their *in vitro* antibacterial activity against MRSA, MRSE, E.coli, and S.agalactia by agar dilution method according to the National Committee for Clinical Laboratory Standards (NCCLS), 1997. Minimum inhibitory concentration (MIC) is defined as the minimum concentration of the compound required to completely inhibit the bacterial growth. The determination of MIC values was performed in triplicate at pH 7.40. The MICs of the synthesized compounds **5a–f** along with pleuromutilin and tiamulin which were used as reference drugs are depicted in Table 1. The MICs of new pleuromutilin derivatives *in vitro* against MRSA, MRSE, E.coli, and S.agalactia ranged from 4 to 0.25 μg/mL, 32 to 1 μg/mL, 32 to 4 μg/mL, and 16 to 1 μg/mL respectively.

**Table 1:** MIC (μg/mL)of 5a–f for MRSA, MRSE, E. coli and S.agalactia.

Compound	MRSA	MRSE	E. coli	S.agalactia
**5a**	2	16	8	16
**5b**	0.25	1	4	1
**5c**	2	16	16	8
**5d**	2	8	16	4
**5e**	4	32	32	16
**5f**	4	16	32	8
**pleuromutilin**	4	16	32	8
**Tiamulin**	0.5	2	2	2

Antibacterial activity for all the synthesized compounds was evaluated against the above mentioned four bacterial strains. Oxford cup assay was carried out and the zones of inhibition for different concentrations of the synthetic compounds were measured. Data are reported as diameters of growth inhibition (mm) and the results are given in Table 2. Also pleuromutilin and tiamulin were used as reference drugs.

**Table 2:** Zone of Inhibition of 5a–f for MRSA, MRSE, E. coli and S.agalactia (in mm).

Compound	MRSA (μg/mL)		MRSE (μg/mL)		E. coli (μg/mL)		S.agalactia (μg/mL)	
	320	160	320	160	320	160	320	160
**5a**	16.83	15.49	13.57	11.83	13.65	12.06	12.38	11.69
**5b**	19.32	17.52	18.52	17.45	17.02	16.32	17.70	16.05
**5c**	16.20	15.73	13.34	12.37	12.77	11.58	13.45	12.48
**5d**	17.06	15.81	13.47	12.36	12.54	11.08	15.79	13.86
**5e**	15.28	13.65	12.36	11.45	11.01	10.65	11.60	11.32
**5f**	13.95	13.74	11.67	10.85	11.12	10.55	12.88	11.39
**pleuromutilin**	14.78	14.01	13.86	11.93	12.56	11.71	13.51	12.37
**Tiamulin**	18.02	17.15	16.42	15.38	18.22	16.78	13.33	11.87

Among all the pleuromutilin derivatives examined, compound **5b** showed the highest antibacterial activities than the other synthesized compounds and the two reference drugs. Three compounds, **5a**, **5c** and **5d**, showed moderate antibacterial activity and displayed superior or similar antibacterial activities to those of pleuromutilin but lower antibacterial activities than tiamulin as indicated by MIC values and the zones of inhibition.

From MIC values and the zones of inhibition it was observed that replacement of the diethyl of tertiary amine (**5b)** with isopropyl (**5a**) or dipropyl (**5c**) resulted in lower antibacterial activities noticeably. While replacement of the piperidine of acetamide (**5d)** with pyrrolidine (**5e**) or morpholine (**5f**) resulted in lower antibacterial activities. Also it was observed that the compounds with straight-chain alkanes in the tertiary amine preferentially showed higher activities than compounds with cycloalkanes in the tertiary amine.

### Molecular Docking Study

In view of their biological activities and structural diversity, the synthesized compounds **5a–f** were subjected to molecular docking study. A PTC ribosome model based on the X-ray structure of *Deinococcus radiodurans* in complex with tiamulin [24] was constructed that consists of all residues 30 Å from the PTC binding site. The docking experiments were performed with Homdock software in Chil^2^ package [25]. Dock binding affinities of those compounds were evaluated according to many parameters including: the binding free energies (ΔG_b_, kcal/mol), hydrogen bonding or other noncovalent molecular interaction, and RMSD values in comparison to the native co-crystallized ligand. The lowest binding free energies and the lowest RMSD values were considered as the best fitted ones [26]. Test docking calculations using tiamulin (Figure 1) were carried out to compare experimental and predicted binding modes and validate our docking protocol. The best tiamulin docking pose agreed well with the experimental binding mode with RMSD of 0.99.

**Figure 3:**
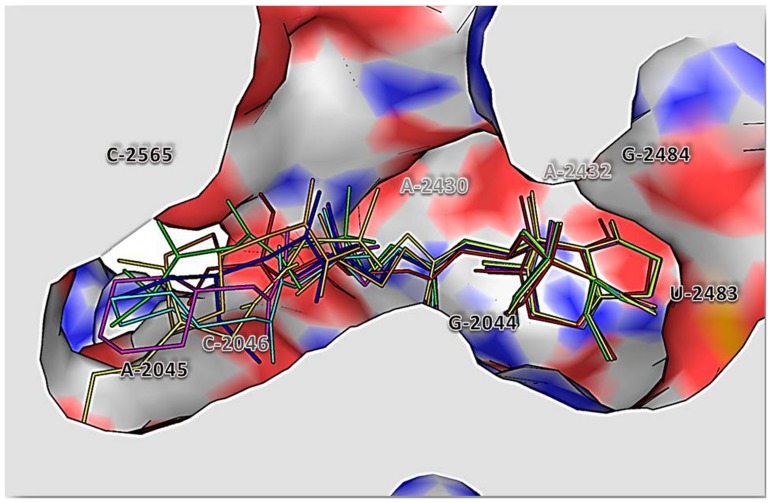
Results of the docking of 5a (green), 5b (blue), 5c (yellow), 5d (magenta), 5e (cyan), 5f (orange), and native ligand tiamulin (red) into the PTC model binding site. The 50S subunit of *Deinococcus radiodurans* cut in half to reveal the binding site.

**Figure 4:**
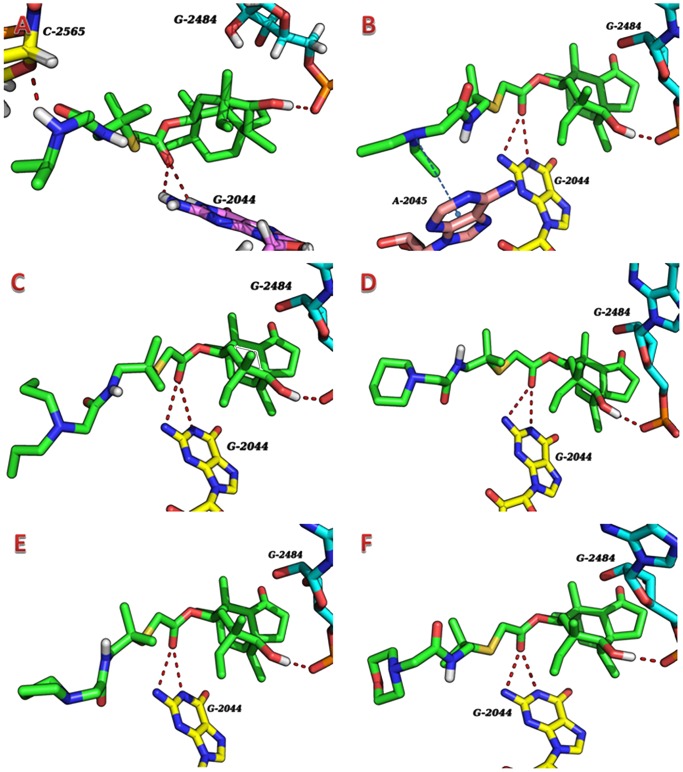
Docking of compounds 5a-f to 1XBP. The compounds 5a-f are colored by green. The hydrogen bonds are shown as red dashed lines and the cation–π interaction is shown as blue dashed lines.

The calculations with the flexible docking protocol placed the six compounds correctly into the binding pocket and the docking results revealed a similar binding pattern as presented in Figure 3, which shows a superposition of the six docked compounds and the tiamulin, a native ligand which is embedded in cocrystallized ribosome. The docking results reveal the binding free energies (ΔG_b_) being in the range of −11.90 to −13.42 kcal/mol, with RMSD range of 0.94 to 1.12 Å. The hydrogen bonding plays an important role in the binding of compounds and 1XBP. As shown in Table S1 and Figure 4, all the six compounds are found to bind with the same hydrogen bondings formed between the hydroxyl group of eight-membered ring and residue of G-2484, and between the ester of side chain and residue of G-2044. Moreover, a cation–π interaction formed between tertiary amine of **5b** and A-2045 play an important role in increasing the binding affinity. The geometry of the interaction further, confirming that cation-π interactions are strongest when the cation is situated perpendicular to the plane of atoms [27]. We presume that the conformation of **5b** make its tertiary amine perpendicular to the purine ring of A-2045. Although compounds **5c-f** also bear a tertiary amine at the terminal of side chain, no cation–π interaction is found by PoseView, a software tool that can automatically create two-dimensional diagrams of complexes with known 3D structure according to the docking results [28].

### X-ray Crystallographic Study of Intermediate 4

Compound **5a-f** were all synthesized starting from intermediate **4**, so its crystallographic structure is necessary and usefull to understand the approximate structures and 3D conformations of compounds **5a-f** in the molecular modeling.

**Figure 5:**
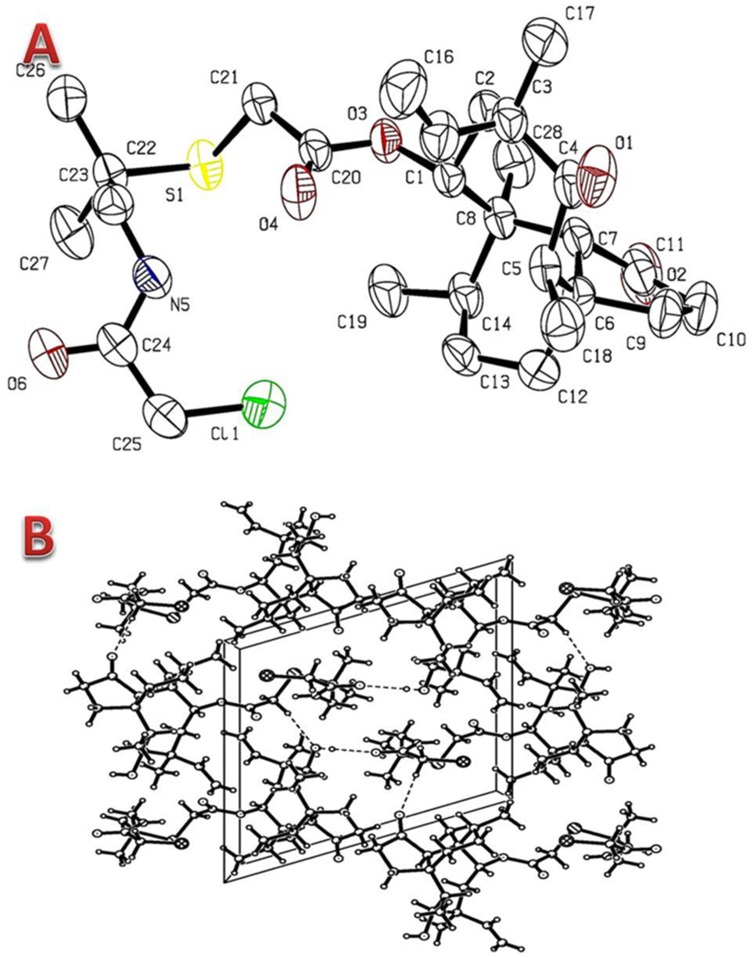
Crystal structure of compound 4. (a) ORTEP diagram for compound **4** with ellipsoids set at 50% probability (hydrogen atoms were omitted for clarity). (b) A perspective view of the molecular packing of **4** viewed along the α axis.

Single crystal of X-ray diffraction study was carried out on the intermediate **4** to understand the nature of its conformational and molecular assembly. Intermediate **4** forms clear light colorless block shaped crystal from a solution of acetone and ethanol by slow evaporation method at room temperature. The crystal structure of **4** was built up of C_28_H_44_ClNO_5_S molecules containing a 5–6–8 tricyclic carbon skeleton, in which all bond lengths and angles were in normal ranges. The crystalline displayed a monoclinic symmetry and the p 1211 space group. The five-membered ring (C(6), C(7), C(9) ∼C(11)) is not planar and the dihedral angles formed by C(6) –C(9) –C(10) and C(6) –C(7) –C(11) is 43.727. The eight-membered ring (C(1), C(2) ∼C(8)) exhibits a boat conformation, while the six-membered ring (C(6), C(7), C(8) C(14), C(13) and C(12)) exhibits a chair conformation. The crystalline conformations of tricyclic carbon skeleton of **4** are very similar to that of the reported pleuromutilin derivative: 14-O-[(3-chlorobenzamide-2-methylpropane-2-yl) thioacetate] Mutilin [29], Perspective views of the title molecules with atomic numbering scheme are shown in Figure 5A, and its packing diagrams are depicted in Figure 5B.

The synthesized chloroacetamide and 2-methylpropane moiety exhibits zig-zag conformation. The crystal is formed by three different intermolecular H-bonds, namely O(1)-H(1)···O(6)^i^, C(21)-H(21B)···O(1)^ii^ and C(25)-H(25A)···O(2)^iii^ (i: −1+x, y, z; ii: −x, −1/2+y, 1-z; iii: −x, 1/2+y, −z), with d (D···A)  = 2.880(4) Å, 3.412(3) and 3.402(4) Å, respectively (hydrogen bond information see in Table S2). Those intermolecular interactions/H-bonds link the molecules into an infinite 3-dimensional supramolecular network structure and play key roles in stabilizing the crystal packing.

## Materials and Methods

### Chemistry

All reagents were purchased from Aladdin (China) and used without further purification. All compounds were synthesized in our lab and identified by IR, NMR and HRMS. Melting points were determined on a Tianda Tianfa YRT-3 apparatus (China) with open capillary tubes and are uncorrected. IR spectra were obtained on a Thermo Nicolet NEXUS-670 spectrometer and recorded as KBr pellets and absorptions are reported in cm^−1^. NMR spectra were recorded using Bruker- 400 MHz spectrometers in appropriate solvents. Chemical shifts (δ) were expressed in parts per million (ppm) relative to the tetramethylsilane. ^13^C NMR spectra were recorded on 100 MHz spectrometers.High-resolution mass spectra (HRMS) were determined on a Bruker Daltonics APEX II 47e mass spectrometer equipped with an electrospray ion source. All reactions were monitored by TLC on 0.2 mm thick silica gel GF254 pre-coated plates. After elution, plate was visualized under UV illumination at 254 nm for UV active materials. Further visualized was achieved by staining with 0.5% phosphomolybdic acid. Column chromatography was carried out on silica gel (200–300 mesh). The products were eluted in appropriate solvent mixture under air pressure. Concentration and evaporation of the solvent after reaction or extraction was carried out on a rotary evaporator.

#### Synthesis of 14-O-(p-toluene sulfonyloxyacetyl) mutilin 2

14-O-(p-toluene sulfonyloxyacetyl) mutilin **9** were synthesized as described previously [23]. mp: 147–148°C. It was used in the next step without further purification. IR (KBr): 3446, 2924, 2863, 1732, 1633, 1597, 1456, 1371, 1297, 1233, 1117, 1035, 832, 664, 560 cm^−1^. ^1^H NMR (400 MHz, CDCl_3_) δ 0.63 (d, 3H, J = 6.8 Hz), 0.87 (d, 3H, J = 6.8Hz), 1.11–1.15 (m, 1H), 1.22–1.26 (s, 5H), 1.33–1.36 (m, 1H), 1.41–1.44 (m, 1H), 1.46–1.50 (m, 5H), 1.63–1.65 (dd, 2H,J_1_ = 10Hz,J_2_ = 7.2 Hz), 2.01–2.08(m, 3H), 2.21–2.29 (m, 3H), 2.45(s, 3H), 3.34 (d, 1H, J = 6.4 Hz), 4.48 (s, 2H), 5.17–5.21 (d, 1H, J = 8.8 Hz), 5.31–5.34 (d, 1H, J = 6.4 Hz), 5.75–5.78 (d, 1H, J = 4.2 Hz), 6.43(q, 1H, J = 17.2 Hz, 10.8 Hz); 7.35–7.37 (d, 2H, J = 4.0 Hz), 7.80–7.82 (d, 2H, J = 4.0 Hz). ^13^C NMR (100 MHz, CDCl_3_) δ 216.7, 164.8, 145.2, 138.6, 132.5, 129.9, 127.9, 117.2, 74.4, 70.2, 64.9, 57.9, 45.3, 44.4, 43.9, 41.7, 36.4, 35.9, 34.3, 30.2, 26.7, 26.3, 24.7, 21.6, 16.4, 14.6, 11.4. HRMS (ESI) of C_29_H_40_O_7_S [M+H]^+^ calcd, 533.2501; found, 533.2507.

#### Synthesis of 14-O-[(1-amino-2-methylpropane-2-yl) thioacetyl] mutilin 3

14-O-[(1-amino-2-methylpropane-2-yl) thioacetyl] mutilin **10** were synthesized as described previously [20], [23]. mp: 154–155°C; IR (free base, KBr): 3351, 2956, 2864, 1734, 1721, 1634, 1456, 1373, 1274, 1209, 1112, 1033, 982, 955, 941, 916 cm^−1^; ^1^H NMR (400 MHz, CDCl_3_) δ 0.73 (d, 3H, J = 7.2 Hz), 0.87 (d, 3H, J = 7.2 Hz), 1.09–1.16 (m, 1H), 1.23 (s, 6H), 1.30–1.38 (m, 2H), 1.45 (s, 1H), 1.52–1.53 (m, 7H), 1.55–1.60 (m, 1H), 1.63–1.69 (m, 2H), 1.74–1.78(q, 1H, J = 0.8 Hz), 2.04–2.10 (q, 2H), 2.18–2.25(m, 2H), 2.32–2.59 (q, 1H, J = 6.8 Hz), 3.09 (s, 2H), 3.13–3.17 (t, 2H, J = 1.6 Hz), 3.35 (d, 1H, J = 6.4 Hz), 5.17–5.22 (q, 1H, J = 1.6 Hz), 5.31–5.34 (q, 1H, J = 1.2 Hz), 5.74(d, 1H, J = 8.4), 6.44–6.51 (q, 1H, J_1_ = 11.2 Hz, J_2_ = 10.8 Hz); ^13^C NMR(100 MHz, CDCl_3_) δ 216.9, 169.4, 139.0, 117.1, 74.6, 69.3, 58.2, 51.7, 48.5, 45.4, 44.7, 43.9, 41.8, 36.7, 35.9, 34.4, 31.2, 30.4, 26.8, 26.3, 26.2, 24.8, 16.8, 14.9, 11.4. HRMS (ESI) of C_26_H_43_NO_4_S [M+H]^+^ calcd, 466.2986; found, 466.2995.

#### Synthesis of 14-O-[(2-chloroacetamide -2-methylpropane-2-yl) thioacetyl] mutilin 4

To a solution of 14-O-[(1-amino-2-methylpropane-2-yl) thioacetyl] mutilin (1.40 g, 3 mmol) and N-methylmorpholine (0.61 g, 6 mmol) in 20 mL dry DCM, ClCH_2_COCl (0.51 g, 4.5 mmol) in 5 mL dry DCM was slowly dropped at 0°C. The reaction mixture was stirred for 2.5 h. After the reaction, the solution was washed with water three times, and then the organic layer was dried with MgSO_4_, filtered, concentrated, and purified by column chromatography (petroleum ether: ethyl acetate  = 1:1) to yield **4** as a white solid (1.38 g, yield: 85%). mp: 171–173°C; IR (KBr): 3449, 3371, 2977, 2924, 2856, 1738, 1713, 1547, 1456, 1414, 1383, 1285, 1265, 1221, 1131, 1114, 1024, 976, 953, 919, 770, 727, 667, 592 cm^−1^; ^1^H NMR (400 MHz, CDCl_3_) δ 7.45, 6.44 (dd, *J* = 17.4, 11.0 Hz, 1H), 5.71 (d, *J* = 8.4 Hz, 1H), 5.27 (d, *J* = 11.0 Hz, 1H), 5.16 (d, *J* = 17.4 Hz, 1H), 4.05 (s, 2H), 3.47–3.06 (m, 5H), 2.50–1.94 (m, 5H), 1.69 (dd, *J* = 35.6, 13.1 Hz, 2H), 1.63–1.17 (m, 15H), 1.17–0.90 (m, 4H), 0.85 (d, *J* = 6.9 Hz, 3H), 0.69 (d, *J* = 6.9 Hz, 3H). ^13^C NMR (101 MHz, CDCl_3_) δ 216.77, 169.79, 166.12, 139.01, 117.13, 74.56, 69.98, 58.10, 47.79, 46.89, 45.43, 44.87, 43.98, 42.68, 41.79, 36.69, 36.01, 34.41, 31.50, 30.40, 29.64, 26.87, 26.12, 24.82, 16.88, 14.86, 11.47. HRMS (ESI) of C_28_H_44_ClNO_5_S [M+Na]^+^ calcd, 564.2521; found, 564.2526.

#### General procedure for the synthesis of compounds 15a–f

Secondary amines (4.5 mmol) was added to the solutions of compound **4** (1.63 g, 3 mmol) and triethylamine (0.61 g, 6 mmol) in tetrahydrofuran (60 mL) and stirred at 45°C for the specified time. Then the tetrahydrofuran was evaporated in vacuum from the reaction mixture. The residue was added ethyl acetate (60 mL) and quenched with saturated aqueous NH_4_Cl (30 mL). The organic layer was separated, washed with water (20 mL for three times), dried with anhydrous Na_2_SO_4_ and rotary evaporated to dryness. Crude residue was purified over silica gel column chromatography afford the desired compounds.

#### 14-O-[(2- isopropylaminoacetyl −2-methylpropane-2-yl) thioacetate]Mutilin (5a)

Compound **5a** was prepared according to the general procedure with a reaction time of 3.5 hours. The crude product was purified over silica gel column chromatography (petroleum ether: ethyl acetate  = 1∶1.5) yielding **5a** (69%, 1.17 g) as a white solid. mp: 105–107°C; IR (KBr): 3437, 2933, 1732, 1668, 1538, 1456, 1416, 1384, 1283, 1152, 1117, 1017, 981, 939, 916 cm^−1^; ^1^H NMR (400 MHz, CDCl_3_) δ 7.69 (s, 1H), 6.42 (dd, *J* = 17.2, 11.2 Hz, 1H), 5.73 (d, *J* = 8.0 Hz, 1H), 5.27 (d, *J* = 10.4 Hz, 1H), 5.16 (d, *J* = 17.3 Hz, 1H), 3.38–3.12 (m, 6H), 2.63 (s, 4H), 2.38–2.14 (m, 3H), 2.12–2.00 (m, 2H), 1.76 (d, *J* = 24.6 Hz, 5H), 1.60 (dd, *J* = 19.4, 8.6 Hz, 3H), 1.51 (d, *J* = 13.5 Hz, 1H), 1.43 (s, 4H), 1.28 (dd, *J* = 36.9, 11.4 Hz, 9H), 1.18–1.05 (m, 4H), 0.85 (d, *J* = 6.7 Hz, 3H), 0.70 (d, *J* = 6.7 Hz, 3H). ^13^C NMR (101 MHz, CDCl_3_) δ 216.56, 170.71, 168.97, 138.73, 116.82, 74.29, 69.22, 58.78, 57.90, 54.24, 46.78, 46.63, 45.15, 44.53, 43.63, 41.51, 36.44, 35.77, 34.13, 31.27, 30.14, 26.53, 26.19, 24.55, 23.75, 23.49, 16.50, 14.59, 11.14. HRMS (ESI) of C_31_H_52_N_2_O_5_S [M+H]^+^ calcd, 565.3592; found, 565.3599.

#### 14-O-[(2-(bis (ethyl) amino) acetamido-2-methylpropane-2-yl) thioacetate]Mutilin (5b)

Compound **5b** was prepared according to the general procedure with a reaction time of 3 hours. The crude product was purified over silica gel column chromatography (petroleum ether: ethyl acetate  = 1:1) yielding **5b** (78%, 1.35 g) as a white solid. mp: 142–145°C; IR (KBr): 3444, 2933, 1732, 1667, 1519, 1455, 1417, 1385, 1282, 1207, 1116, 1065, 1021, 981, 953, 916 cm^−1^; ^1^H NMR (400 MHz, CDCl_3_) δ 6.41 (dd, *J* = 16.4, 10.2 Hz, 1H), 5.74 (dd, *J* = 35.0, 7.6 Hz, 1H), 5.27 (s, 1H), 5.15 (d, *J* = 17.3 Hz, 1H), 3.20 (dd, *J* = 60.8, 26.5 Hz, 5H), 2.91 (d, *J* = 85.4 Hz, 1H), 2.70–2.39 (m, 3H), 2.40–1.92 (m, 7H), 1.75 (d, *J* = 24.2 Hz, 3H), 1.56 (dd, *J* = 44.4, 11.4 Hz, 5H), 1.41 (s, 5H), 1.36–1.18 (m, 7H), 1.18–1.00 (m, 6H), 0.95 (t, *J* = 6.6 Hz, 2H), 0.84 (d, *J* = 5.9 Hz, 3H), 0.69 (s, 3H); ^13^C NMR (101 MHz, CDCl_3_) δ 216.90, 169.19, 139.03, 117.09, 74.58, 69.48, 58.20, 54.53, 48.70, 47.67, 47.35, 45.44, 44.82, 43.90, 41.80, 36.76, 36.06, 34.42, 31.56, 30.44, 27.44, 26.91, 26.45, 24.84, 24.04, 16.81, 14.88, 12.55, 11.43. HRMS (ESI) of C_32_H_54_N_2_O_5_S [M+H]^+^ calcd, 579.3826; found, 579.3835.

#### 14-O-[(2-(bis (propyl) amino) acetamido-2-methylpropane-2-yl) thioacetate]Mutilin (5c)

Compound **5c** was prepared according to the general procedure with a reaction time of 3 hours. The crude product was purified over silica gel column chromatography (petroleum ether: ethyl acetate  = 1:1) yielding **5c** (62%, 1.13 g) as a white solid. mp: 102–104°C; IR (KBr): 3444, 2931, 1732, 1667, 1456, 1417, 1384, 1281, 1152, 1117, 1019, 981, 953, 916 cm^−1^; ^1^H NMR (400 MHz, CDCl_3_) δ 6.53–6.34 (m, 1H), 5.75 (dd, *J* = 30.4, 4.7 Hz, 1H), 5.35–5.11 (m, 2H), 4.48 (dd, *J* = 54.5, 18.2 Hz, 1H), 3.60–3.44 (m, 1H), 3.31 (dd, *J* = 34.3, 15.3 Hz, 3H), 3.26–3.08 (m, 2H), 2.81 (d, *J* = 3.2 Hz, 1H), 2.61 (d, *J* = 57.1 Hz, 3H), 2.44–2.16 (m, 5H), 2.03 (dd, *J* = 19.9, 12.3 Hz, 4H), 1.96–1.55 (m, 8H), 1.58–1.18 (m, 14H), 1.14 (s, 6H), 0.85 (s, 3H), 0.70 (d, *J* = 2.9 Hz, 3H); ^13^C NMR (101 MHz, CDCl_3_) δ 216.86, 171.42, 168.94, 138.97, 117.08, 74.55, 69.38, 58.15, 55.99, 53.86, 51.88, 49.36, 47.62, 45.38, 45.30, 44.75, 43.87, 41.74, 36.71, 35.99, 34.37, 30.38, 26.78, 26.40, 24.79, 23.75, 17.59, 16.79, 14.85, 11.39. HRMS (ESI) of C_34_H_58_N_2_O_5_S [M+H]^+^ calcd, 607.4071; found, 607.4077.

#### 14-O-[(2-(piperidine-1-yl) acetamido-2-methylpropane-2-yl) thioacetate]Mutilin (5d)

Compound **5d** was prepared according to the general procedure with a reaction time of 3.5 hours. The crude product was purified over silica gel column chromatography (petroleum ether: ethyl acetate  = 1:1) yielding **5d** (77%, 1.36 g) as a white solid. mp: 175–1781°C; IR (KBr): 3405, 3316, 2933, 2860, 1729, 1652, 1529, 1454, 1425, 1385, 1297, 1191, 1165, 1138, 1024, 983, 953, 916; ^1^H NMR (400 MHz, CDCl_3_) δ 7.94 (s, 1H), 6.47 (dd, *J* = 17.3, 11.0 Hz, 1H), 5.77 (d, *J* = 8.3 Hz, 1H), 5.25 (dd, *J* = 46.4, 14.1 Hz, 2H), 3.36 (s, 1H), 3.25 (dd, *J* = 19.6, 12.5 Hz, 3H), 2.99 (s, 2H), 2.50 (s, 4H), 2.39–2.30 (m, 1H), 2.23 (dd, *J* = 16.6, 8.7 Hz, 2H), 2.14–2.02 (m, 2H), 1.77 (d, *J* = 14.4 Hz, 1H), 1.73–1.59 (m, 6H), 1.59–1.33 (m, 10H), 1.28 (t, *J* = 7.2 Hz, 6H), 1.23–0.95 (m, 5H), 0.88 (d, *J* = 6.8 Hz, 3H), 0.74 (d, *J* = 6.9 Hz, 3H); ^13^C NMR (101 MHz, CDCl_3_) δ 216.88, 169.26, 139.00, 117.18, 74.62, 69.48, 62.25, 58.23, 55.03, 47.02, 46.86, 45.46, 44.85, 43.93, 41.82, 36.76, 36.08, 34.44, 31.59, 30.46, 26.83, 26.55, 26.48, 26.37, 26.19, 24.87, 23.74, 16.83, 14.92, 11.45. HRMS (ESI) of C_33_H_54_N_2_O_5_S [M+H]^+^ calcd, 591.3826; found, 591.3829.

#### 14-O-[(2-( pyrrolidine-1-yl) acetamido-2-methylpropane-2-yl) thioacetate]Mutilin (5e)

Compound **5e** was prepared according to the general procedure with a reaction time of 3.5 hours. The crude product was purified over silica gel column chromatography (petroleum ether: ethyl acetate  = 1:1) yielding **5e** (71%, 1.23 g) as a white solid. mp: 169–173°C; IR (KBr): 3412, 2926, 1731, 1654, 1531, 1456, 1419, 1385, 1288, 1190, 1118, 1023, 982, 953, 916 cm^−1^; ^1^H NMR (400 MHz, CDCl_3_) δ 6.42 (d, *J* = 11.1 Hz, 1H), 5.70 (s, 1H), 5.27 (d, *J* = 9.3 Hz, 1H), 5.16 (d, *J* = 16.7 Hz, 1H), 4.10–3.98 (m, 1H), 3.32 (dd, *J* = 40.3, 17.2 Hz, 2H), 3.16 (dd, *J* = 22.0, 15.0 Hz, 2H), 2.27 (dd, *J* = 26.0, 20.2 Hz, 4H), 2.01 (dd, *J* = 31.7, 22.4 Hz, 3H), 1.68 (dd, *J* = 47.7, 10.9 Hz, 5H), 1.56–1.30 (m, 9H), 1.26 (dd, *J* = 23.8, 8.3 Hz, 5H), 1.19–0.92 (m, 6H), 0.85 (s, 3H), 0.67 (t, *J* = 13.5 Hz, 3H). ^13^C NMR (101 MHz, CDCl_3_) δ 216.83, 169.80, 166.16, 139.16, 138.94, 117.11, 74.56, 69.99, 60.33, 58.14, 47.80, 46.88, 45.43, 44.87, 44.37, 42.67, 41.79, 36.70, 36.01, 34.41, 31.50, 30.40, 26.75, 26.35, 24.82, 16.78, 14.84, 14.17, 11.46. HRMS (ESI) of C_32_H_52_N_2_O_5_S [M+H]^+^ calcd, 577.3670; found, 577.3683.

#### 14-O-[(2-(morpholine-4-yl) acetamido-2-methylpropane-2-yl) thioacetate]Mutilin (5f)

Compound **5f** was prepared according to the general procedure with a reaction time of 3.5 hours. The crude product was purified over silica gel column chromatography (petroleum ether: ethyl acetate  = 1:1.5) yielding **5e** (73%, 1.29 g) as a white solid. mp: 152–154°C; IR (KBr): 3404, 3329, 2962, 2927, 1729, 1655, 1532, 1455, 1424, 1372, 1298, 1191, 1168, 1118, 1024, 984, 954, 916, 875 cm^−1^; ^1^H NMR (400 MHz, CDCl_3_) δ 6.40 (dd, *J* = 17.4, 11.0 Hz, 1H), 5.73 (d, *J* = 8.4 Hz, 1H), 5.25 (d, *J* = 11.1 Hz, 1H), 5.16 (d, *J* = 17.4 Hz, 1H), 3.88–3.62 (m, 5H), 3.39–3.31 (m, 1H), 3.25 (dd, *J* = 14.0, 6.5 Hz, 1H), 3.20–3.10 (m, 3H), 3.01 (s, 2H), 2.69–2.44 (m, 5H), 2.29 (dd, *J* = 13.6, 6.7 Hz, 1H), 2.27–2.14 (m, 2H), 2.16–1.98 (m, 3H), 1.74 (dd, *J* = 14.4, 2.2 Hz, 1H), 1.71–1.56 (m, 4H), 1.56–1.30 (m, 8H), 1.23 (t, *J* = 7.6 Hz, 8H), 1.19–1.03 (m, 5H), 0.85 (d, *J* = 7.0 Hz, 3H), 0.69 (d, *J* = 7.0 Hz, 3H).^13^C NMR (101 MHz, CDCl_3_) δ 216.77, 169.93, 169.33, 139.03, 117.09, 74.56, 69.64, 67.00, 61.83, 58.17, 53.89, 47.03, 46.89, 45.44, 44.90, 43.93, 41.81, 36.70, 36.12, 34.40, 31.59, 26.82, 26.52, 26.50, 26.44, 24.84, 16.78, 14.88, 11.42. HRMS (ESI) of C_32_H_52_N_2_O_5_S [M+H]^+^ calcd, 593.3619; found, 593.3624.

### Antibacterial activity

The minimum inhibitory concentration (MIC) studies were performed on MRSA, MRSE, E. coli, and S. agalactiae which were all separated from the clinic using agar dilution method according to NCCLS. 12800 μg synthesized compounds and pleuromutilin used as a reference drug were weighed accurately and dissolved in about 5 mL ethanol. Then distilled water was added to the solution to 10 mL. Tiamulin fumarate used as another reference drug was dissolved in 10 mL distilled water directly. Then all the solutions were diluted with distilled water by two fold to provide 10 dilutions down to the lowest concentration of 0.625 μg/mL. 2 mL of the 2-fold serial dilutions of each test compound/drug were incorporated into 18 mL hot Mueller-Hintion agar medium, which resulted in the final concentration of each dilutions decreasing tenfold. Inoculum of MRSA, MRSE, E. coli, and S. agalactiae were prepared from blood slants and adjusted to approximate 10^5^–10^6^ CFU/mL with sterile saline (0.90% NaCl). 10 μL amount of bacterial suspension was spotted into Mueller-Hintion agar plates containing serial dilutions of compounds/drugs. The plates were incubated at 36.5°C for 48 h. The MIC is defined as the minimum concentration of compound to give complete inhibition of bacterial growth. The same procedure was repeated in triplicate.

Oxford cup assay was performed to evaluate the rate of inhibition in the growth of bacteria. Inoculum was prepared in 0.9% saline using McFarland standard and spread uniformly on nutrient agar plates. All the compounds were diluted to 320 and 160 μg/mL and the resulting solutions were added to the Oxford cups which were placed at equidistance on the above agar surface. The zone of inhibition for each concentration was measured after 24 h incubation at 37°C.

### Molecular modeling

The crystal structure of 50S ribosomal subunit from *Deinococcus radiodurans* in complex with tiamulin (PDB ID: 1XBP) [24] was used for all simulations with Homdock software in Chil^2^ package [25] combining of a Graph based molecular alignment (GMA) tool and a Monte-Carlo/Simulated Annealing (MC/SA) algorithm based docking (GlamDock) tool.

Molecular docking was performed with Homdock software in Chil2 package [25], which introduced a similarity based docking. In this study, tiamulin was the template for flexible molecular alignment, and the interaction was optimized by GlamDock according to the Chil^2^ Score scoring function based on ChemScore with smooth, improved potential. All the compounds were prepared with Avogadro software [30], including 5000 steps Steepest Descent and 1000 steps Conjugate Gradients geometry optimization based on MMFF94 force field. 50S ribosomal subunit was extracted from crystal structure of 1XBP and transformed to mol2 format. The docking position was set to the binding site of tiamulin. All the compounds were superposed to tiamulin by the GMA, and the placement of compounds were optimized by a MC/SA algorithm in Glamdock according to ChillScore. During docking, the steps of local gradient based minimization was set to 20, the number of MC/SA runs was set to 10, and 500 steps for each MC/SA run. All the other parameters were set to be default.

As a result of calculations we obtained the output files of the acceptor-ligand complex with flexible residues, and the similarity of docked structures was measured by computing the RMSD between the coordinates of the atoms. The binding affinity between compounds and receptor were estimated by Autodock Score. Hydrogen bonds and other interactions were detected by PoseView [28] and all the figures were generated by PyMol 1.5.03 [31].

### X-ray Crystallographic Structure of Compound 4

The colorless single crystals of compound **4** suitable for X-ray structure determination were obtained by slowly evaporating a mixed solvent of acetone and ethanol for about twenty days at room temperature. A single crystal with dimensions of 0.34 mm ×0.32 mm ×0.21 mm was selected and mounted in air onto thin glass fibers. X-ray intensity data were measured at 293 (2) K on an Agilent SuperNova-CCD diffractometer equipped with a mirror-monochromatic Mo*ka* (λ = 0.7107 Å) radiation. A total of reflections were collected in the range of 3.12≤*θ*≤26.37° (index ranges: −16≤*h*≤16, −9≤*k*≤10, −15≤*l*≤16) by using a ω scan mode with 4839 independent ones (*R*_int_  = 0.0157), of which 4361 with *I*>2*σ* (*I*) were consider as observed and used in the succeeding refinements. The structure was refined with SHELXL program [32] by full-matrix least-squares techniques on *F^2^*. The non-hydrogen atoms were refined anisotropically, and hydrogen atoms were determined with theoretical calculations. A full-matrix least-squares refinement gave the final *R* = 0.0373, *wR* = 0.0869 (*w* = 1/[*σ* ^2^ (*F_o_* ^2^)+(0.0348*P*)^2^+0.2946*P*], where *P* =  (*F_o_* ^2^ +2*Fc* ^2^)/3), *S* = 1.039, (*Δρ*)_ma*x*_  = 0.229, and (*Δρ*)_min_  = −0.212 e/Å^3^ (see Table S3). PLATON 1.17 [33] was used for molecular representations and SHELXL [32] was used for packing diagrams.

## Supporting Information

Figure S1 IR, ^1^H and^13^C NMR spectra of compounds **1–5f**.
(PDF)

Table S1 Bind free energy, number of noncovalent molecular interaction and RMSD.
(DOCX)

Table S2 Hydrogen bond lengths (Å) and bond angles (°) of compound **4**.
(DOCX)

Table S3 Crystallographic Data for Intermediate **4**.
(DOCX)
