# Supplementary figures and images for: Chemical Synthesis and Biological Activities of Novel Pleuromutilin Derivatives with Substituted Amino Moiety
Source: PLoS One. 2013 Dec 23;8(12):e82595. doi: 10.1371/journal.pone.0082595 (PMC3871055; doi:10.1371/journal.pone.0082595)

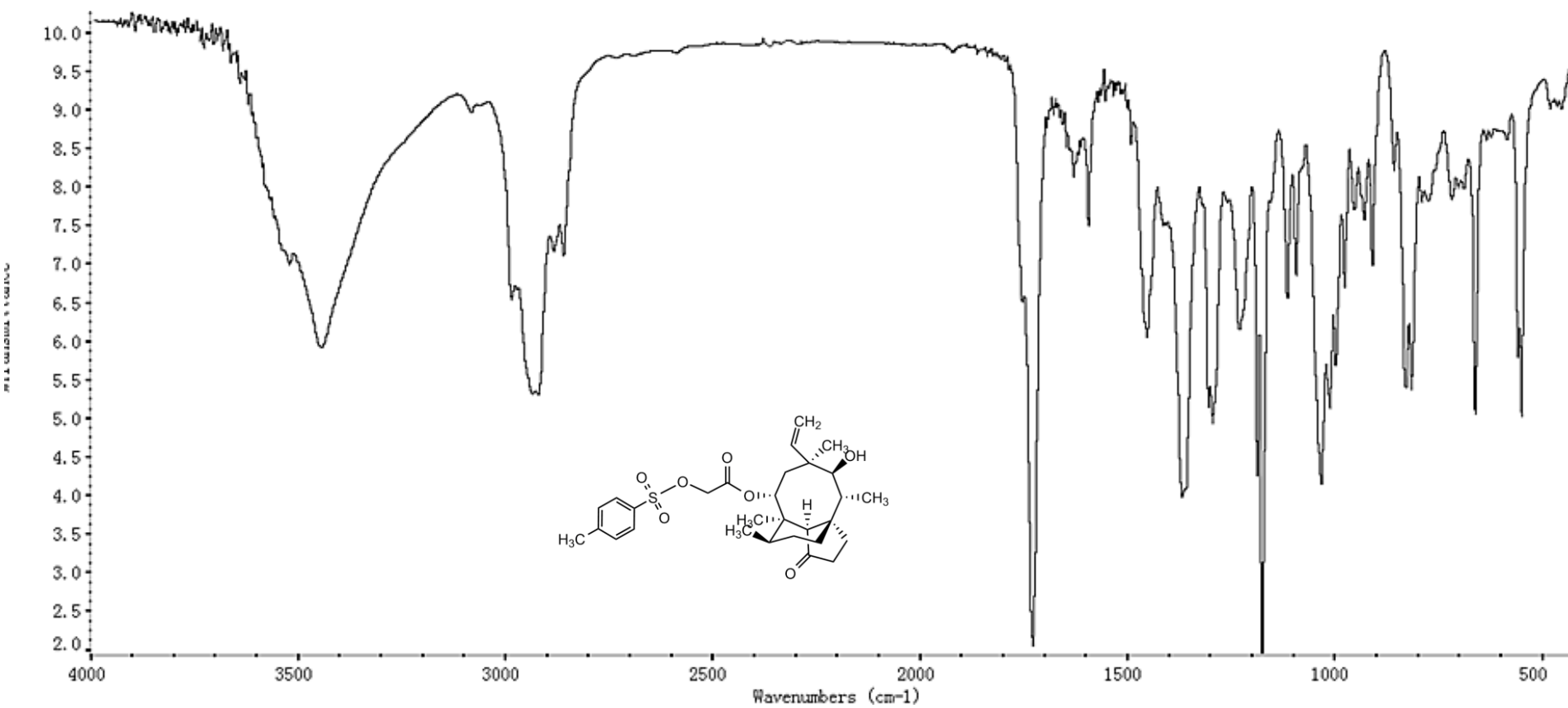



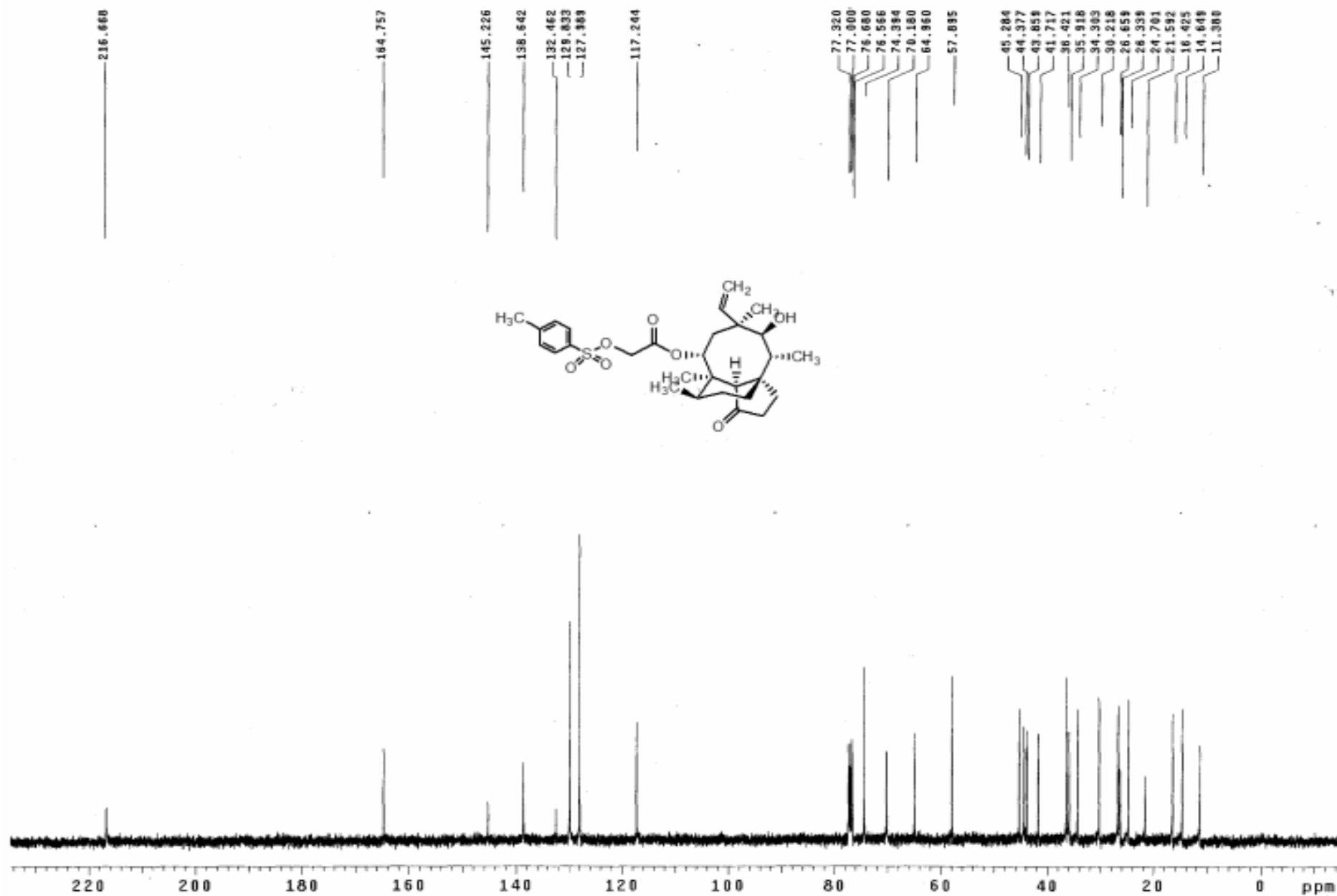

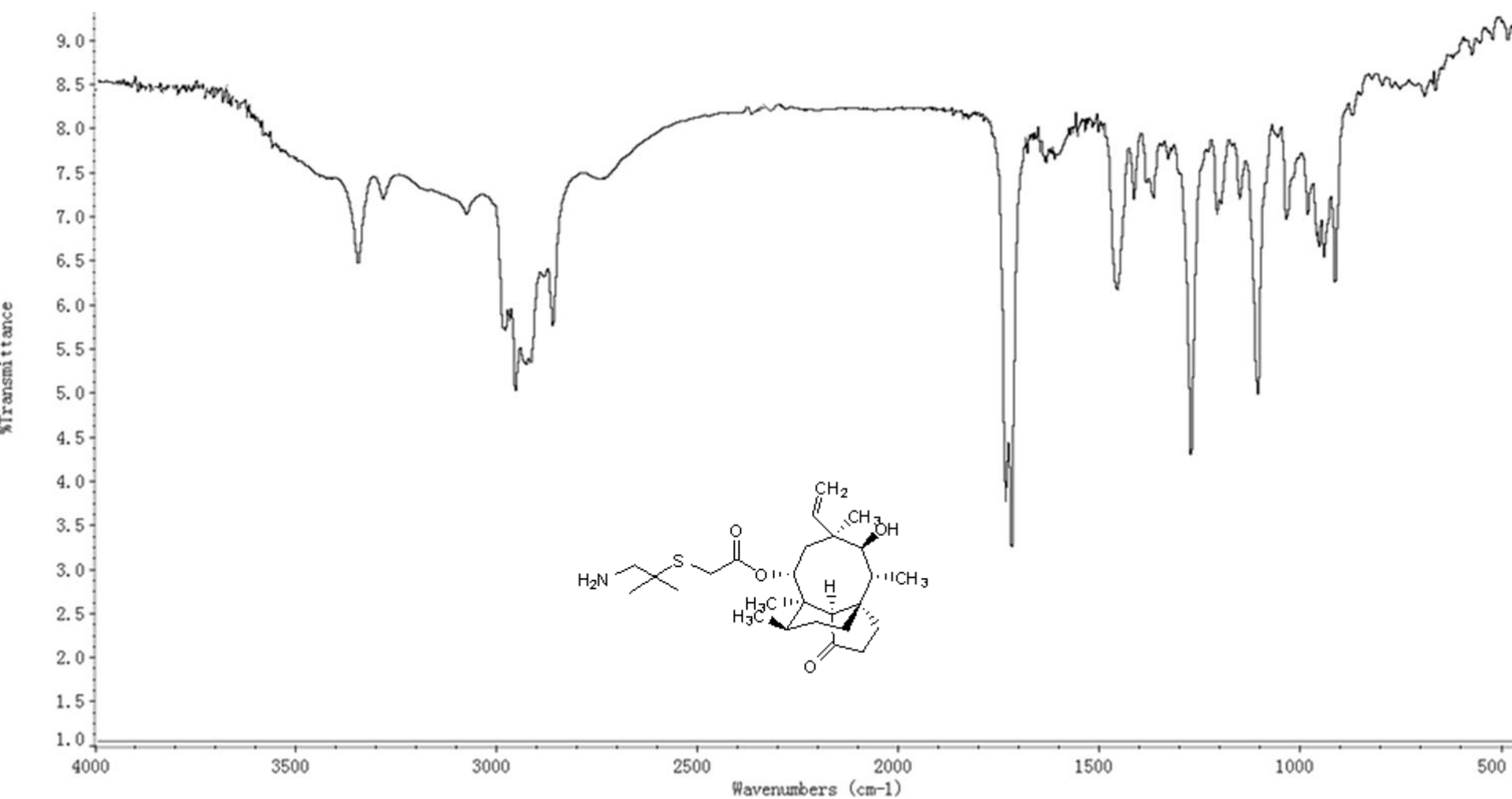

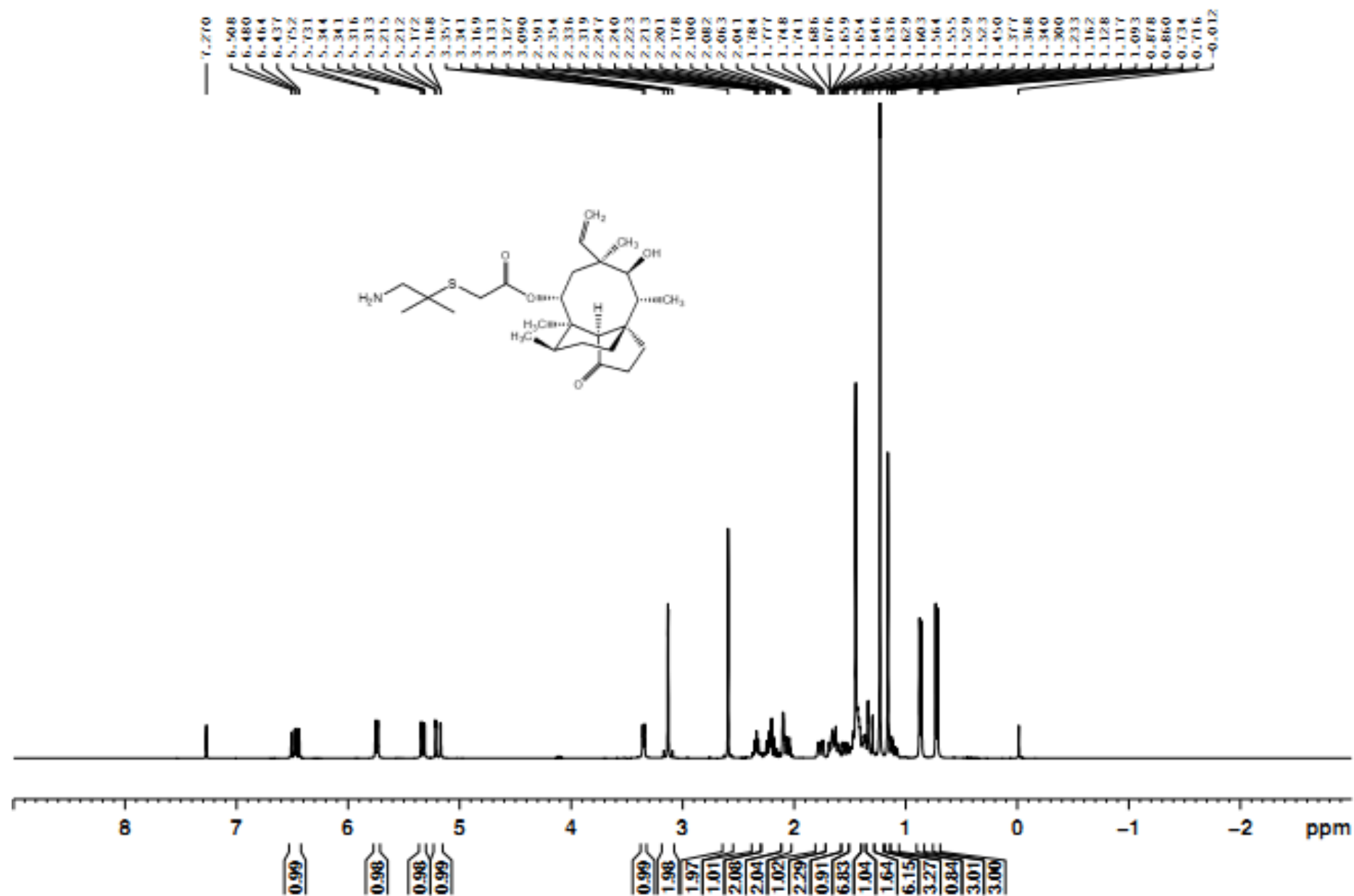

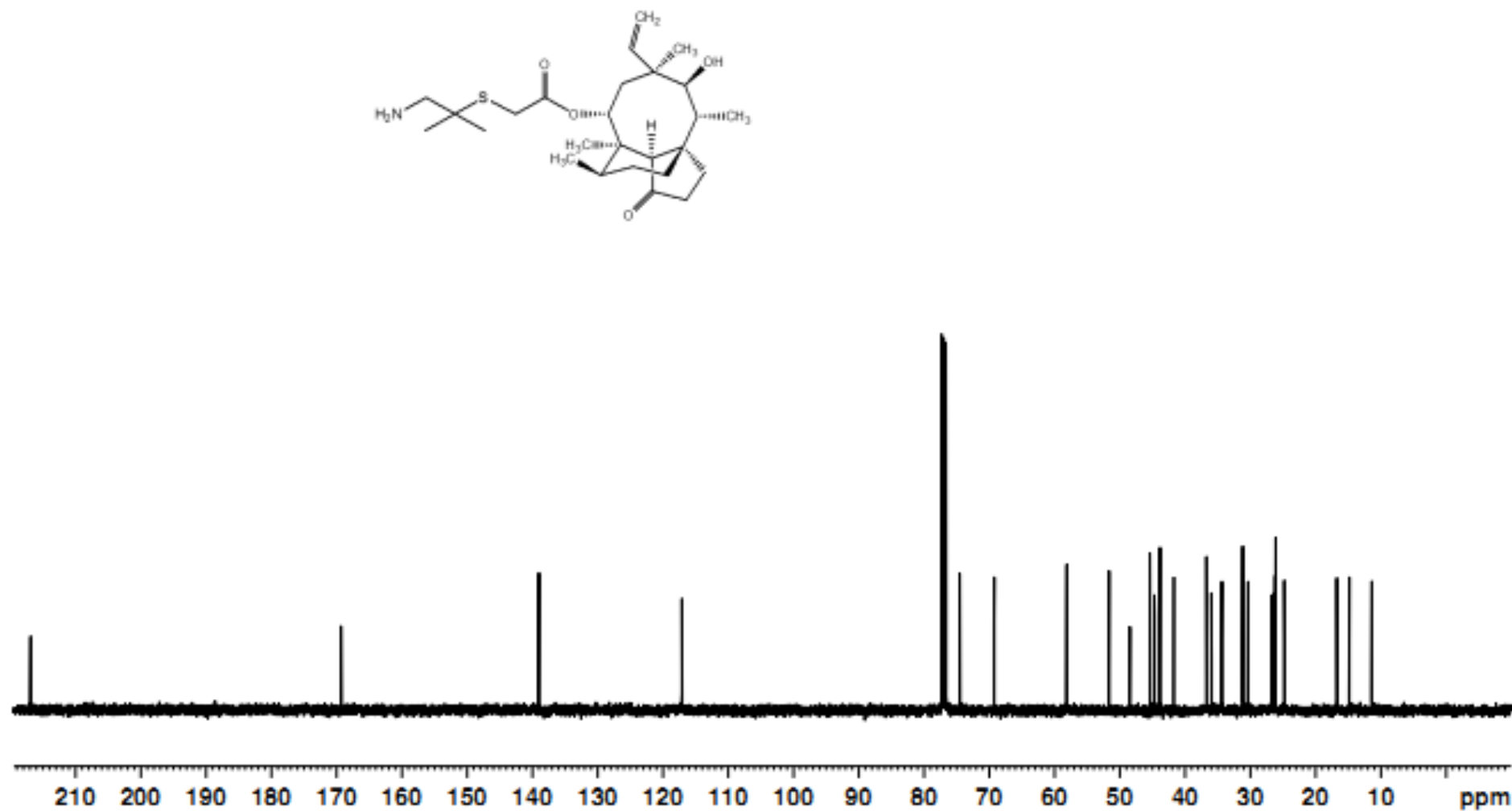

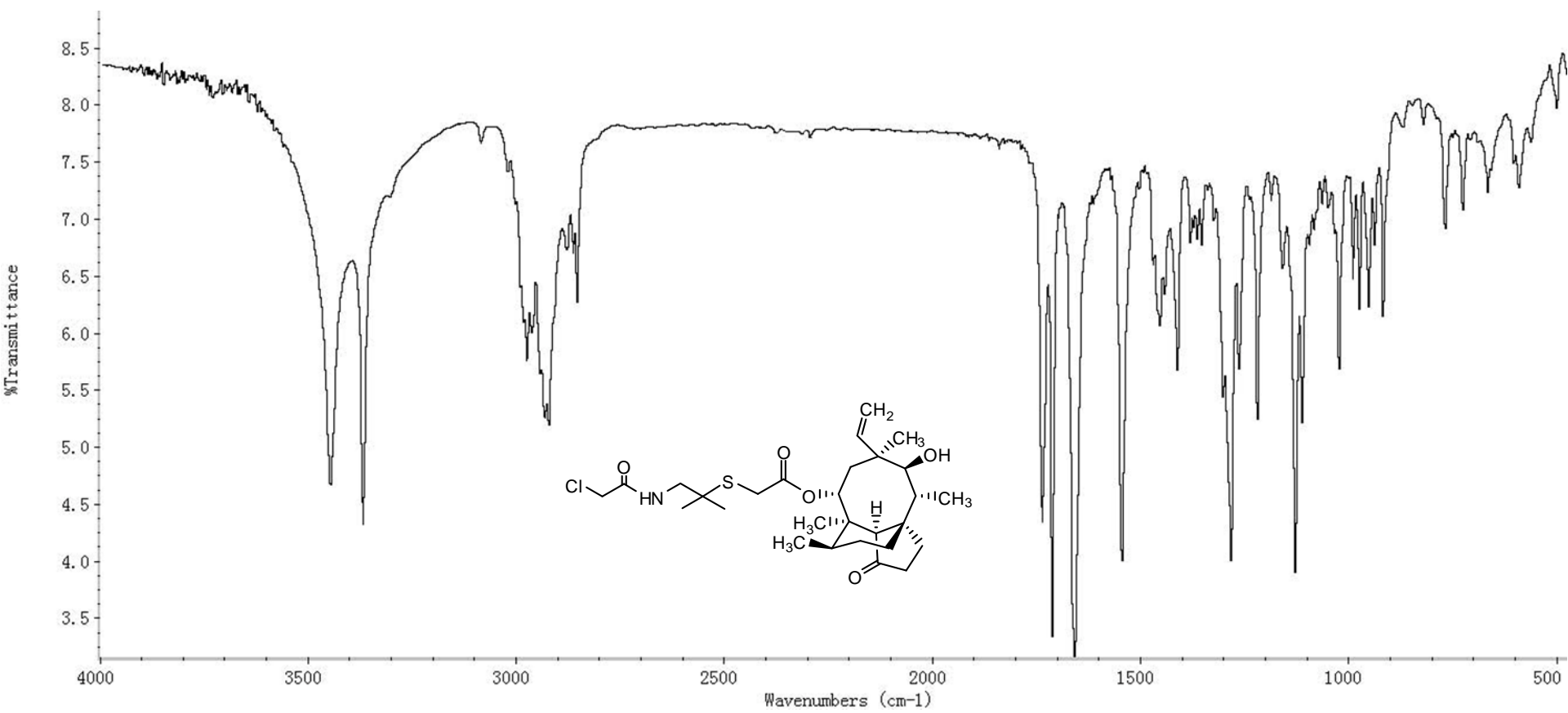

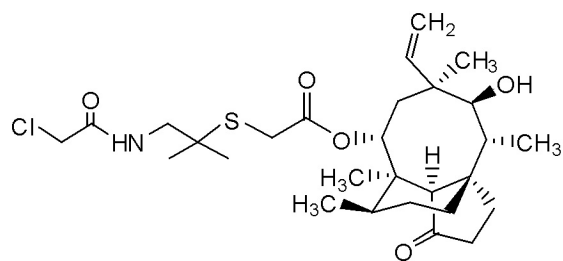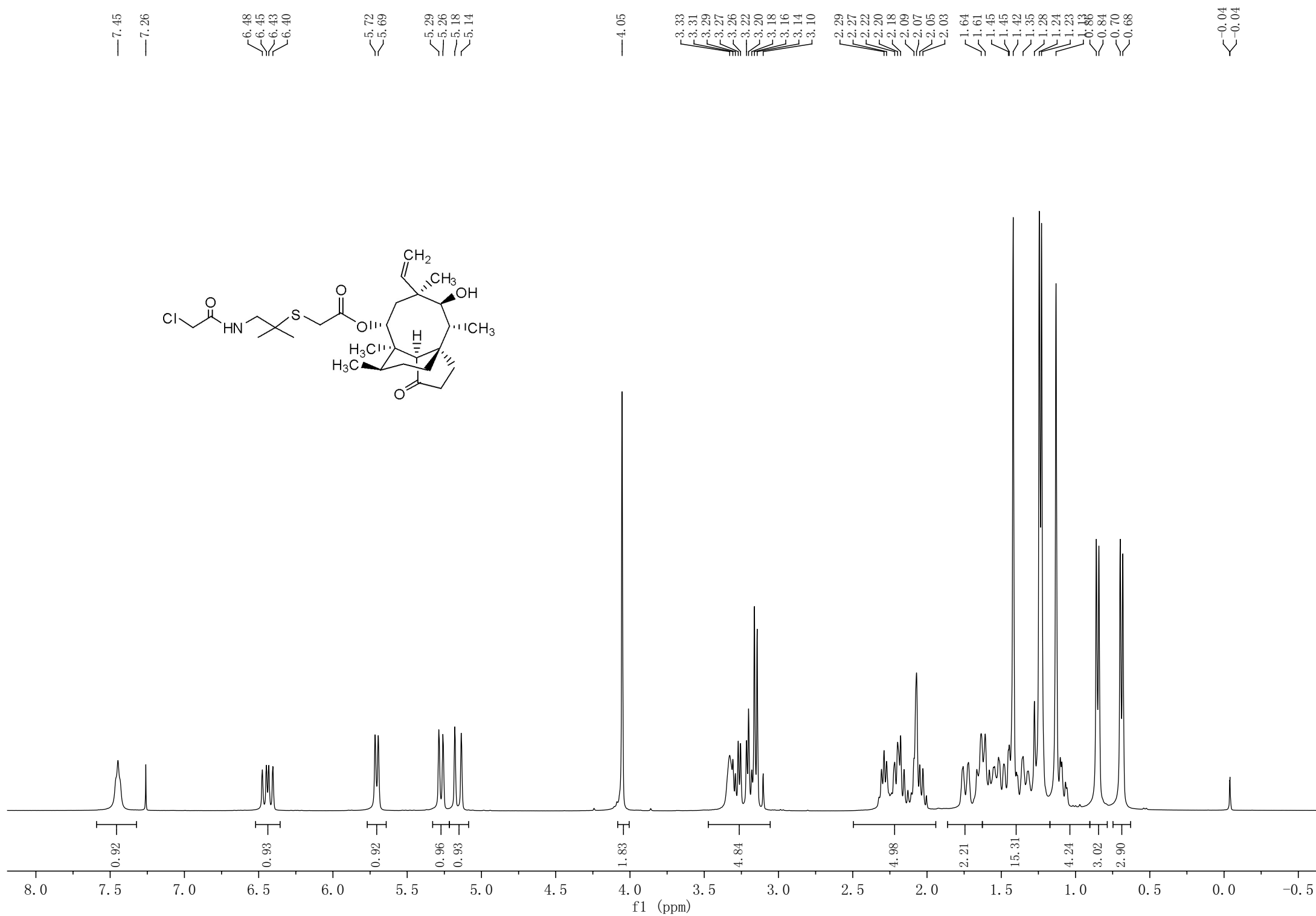

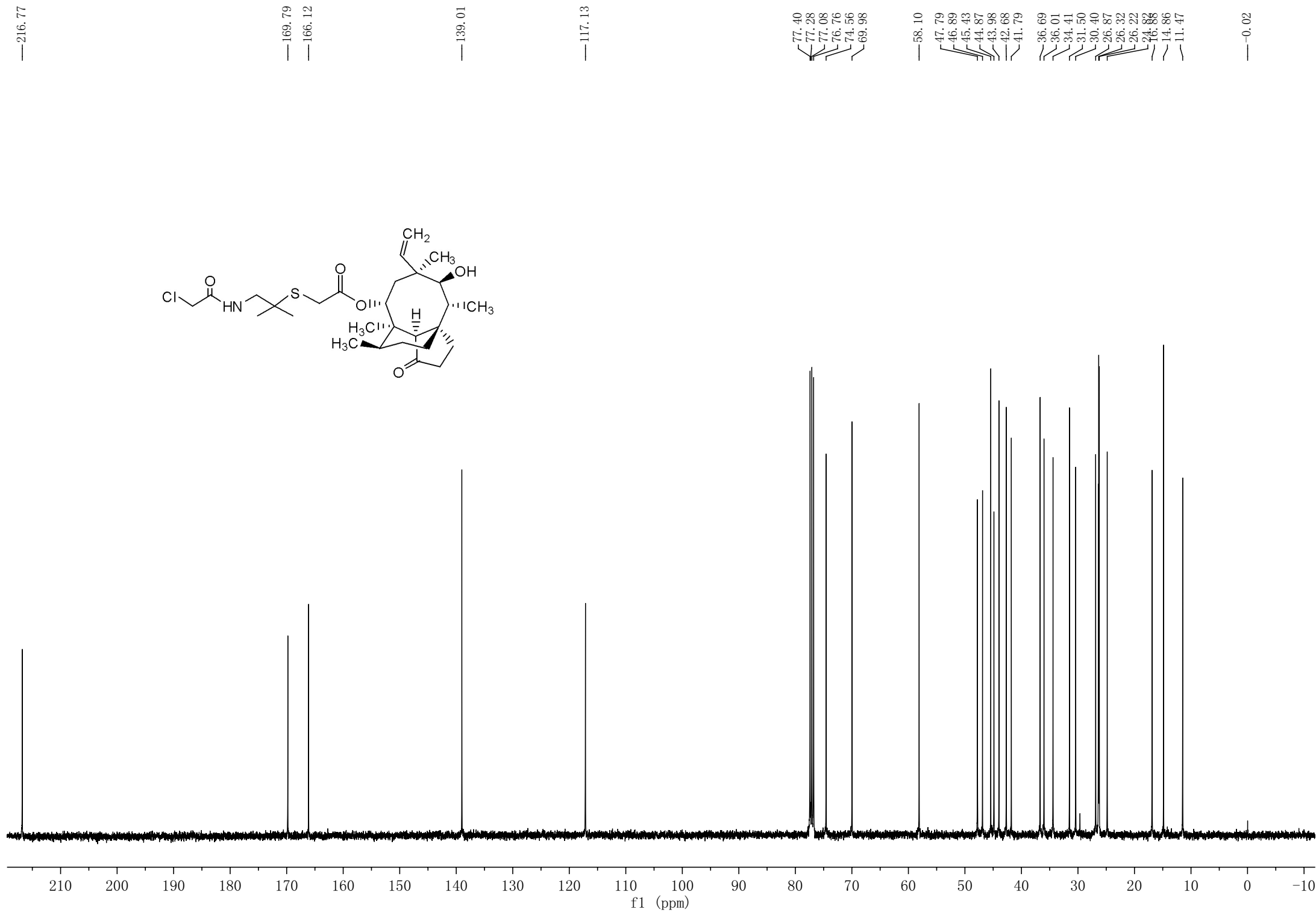

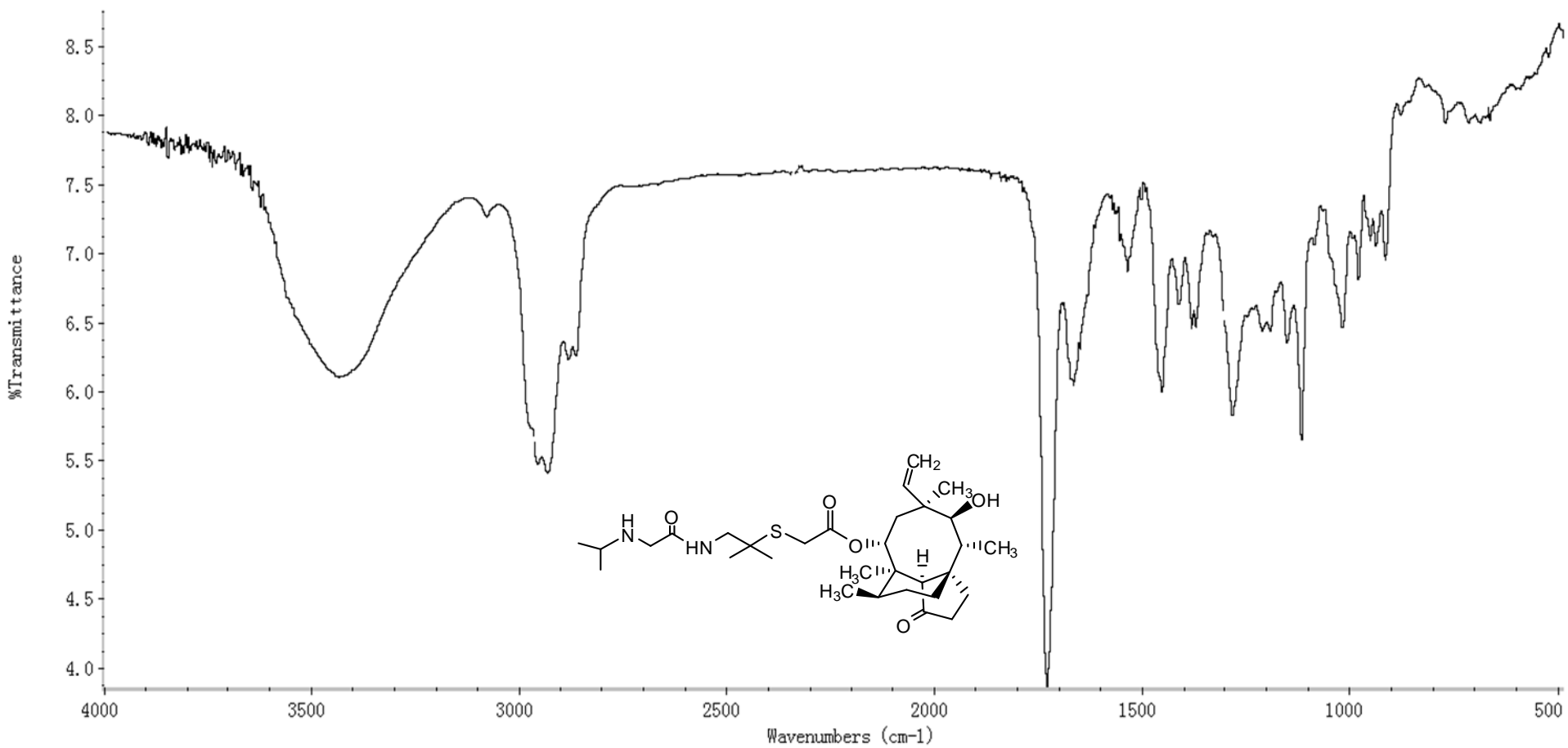

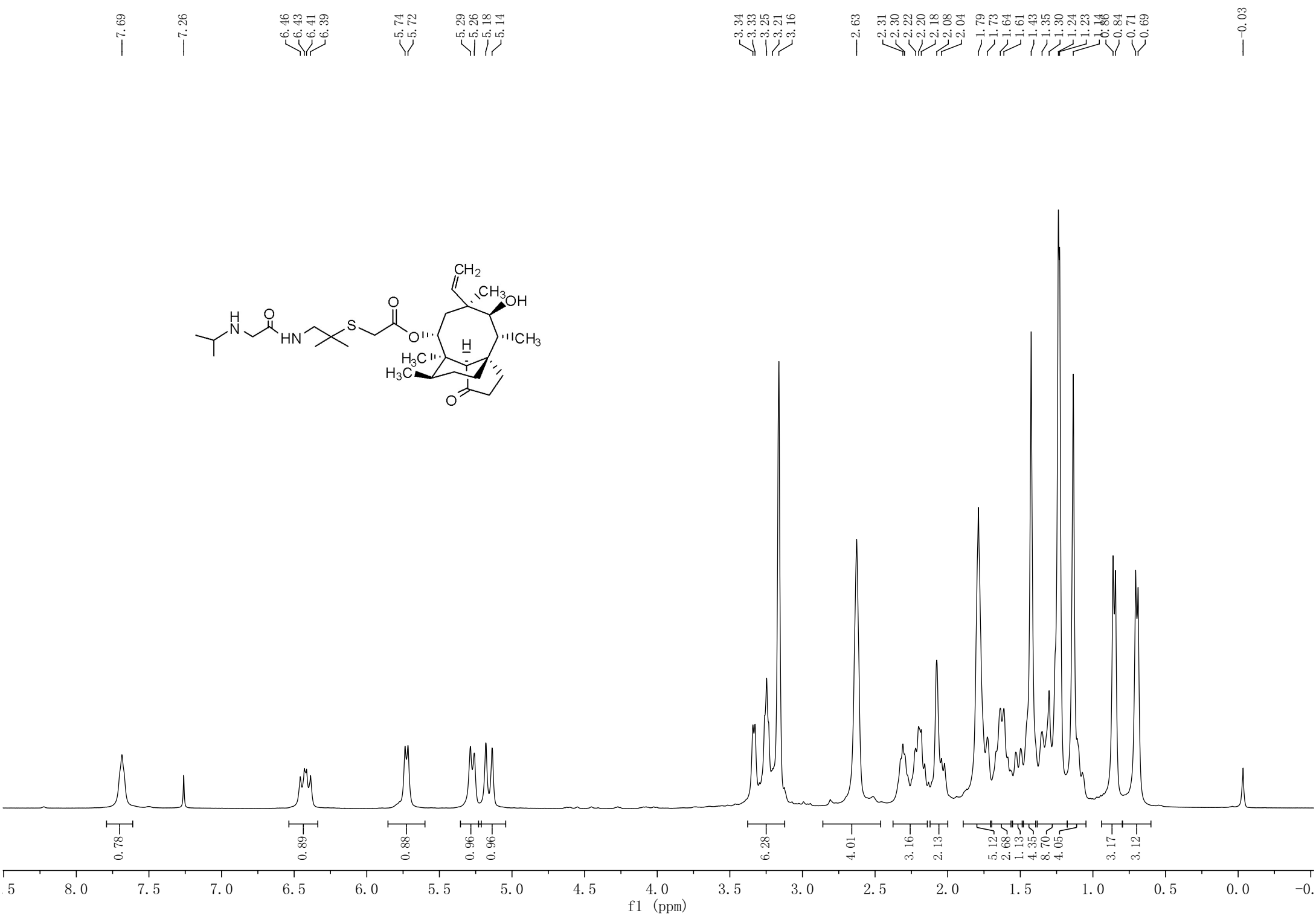

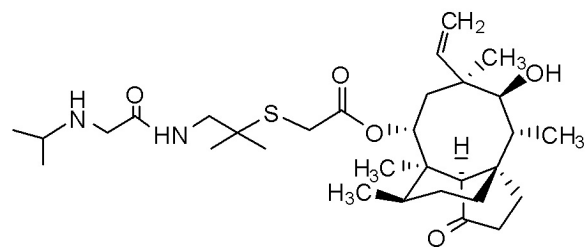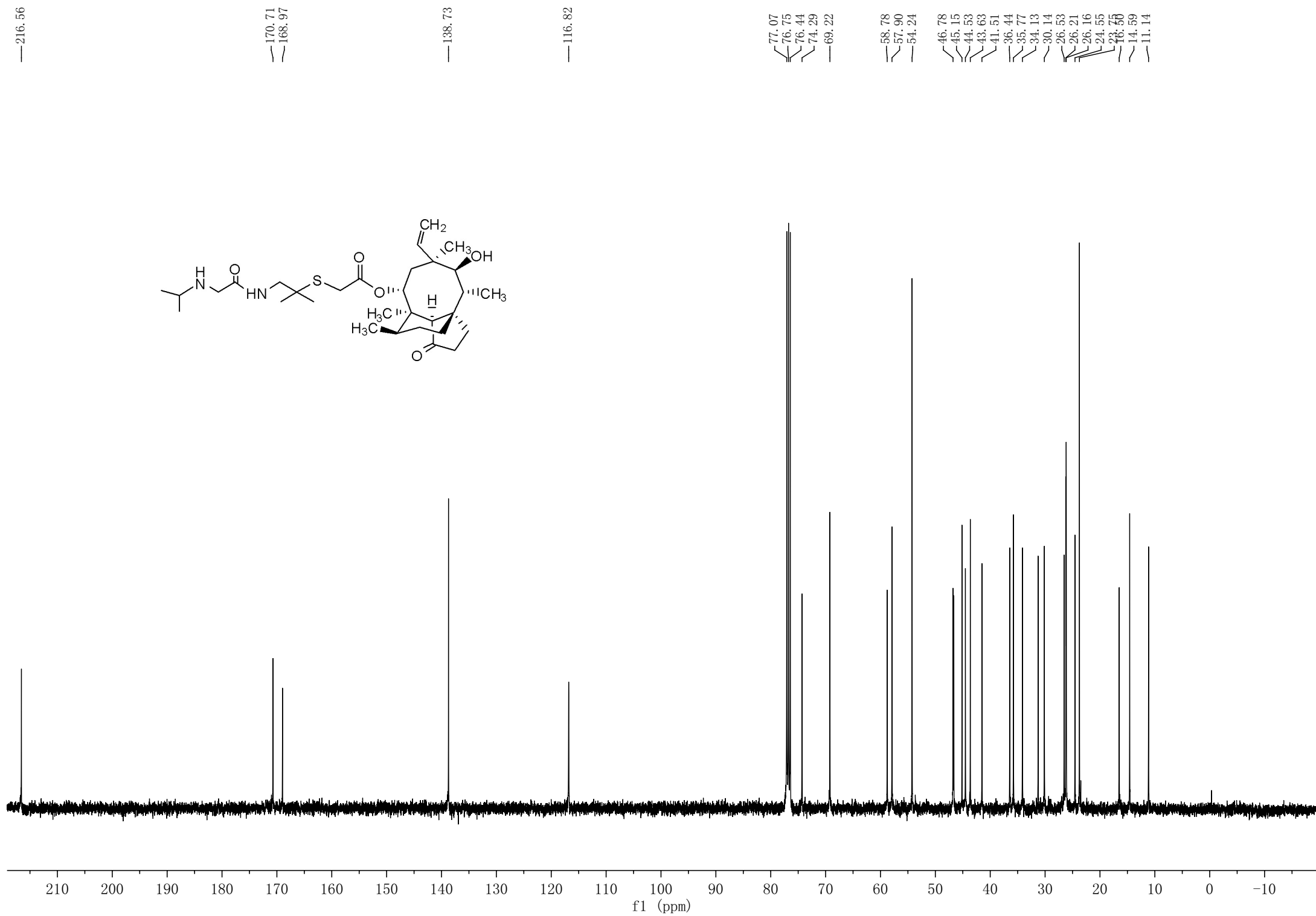

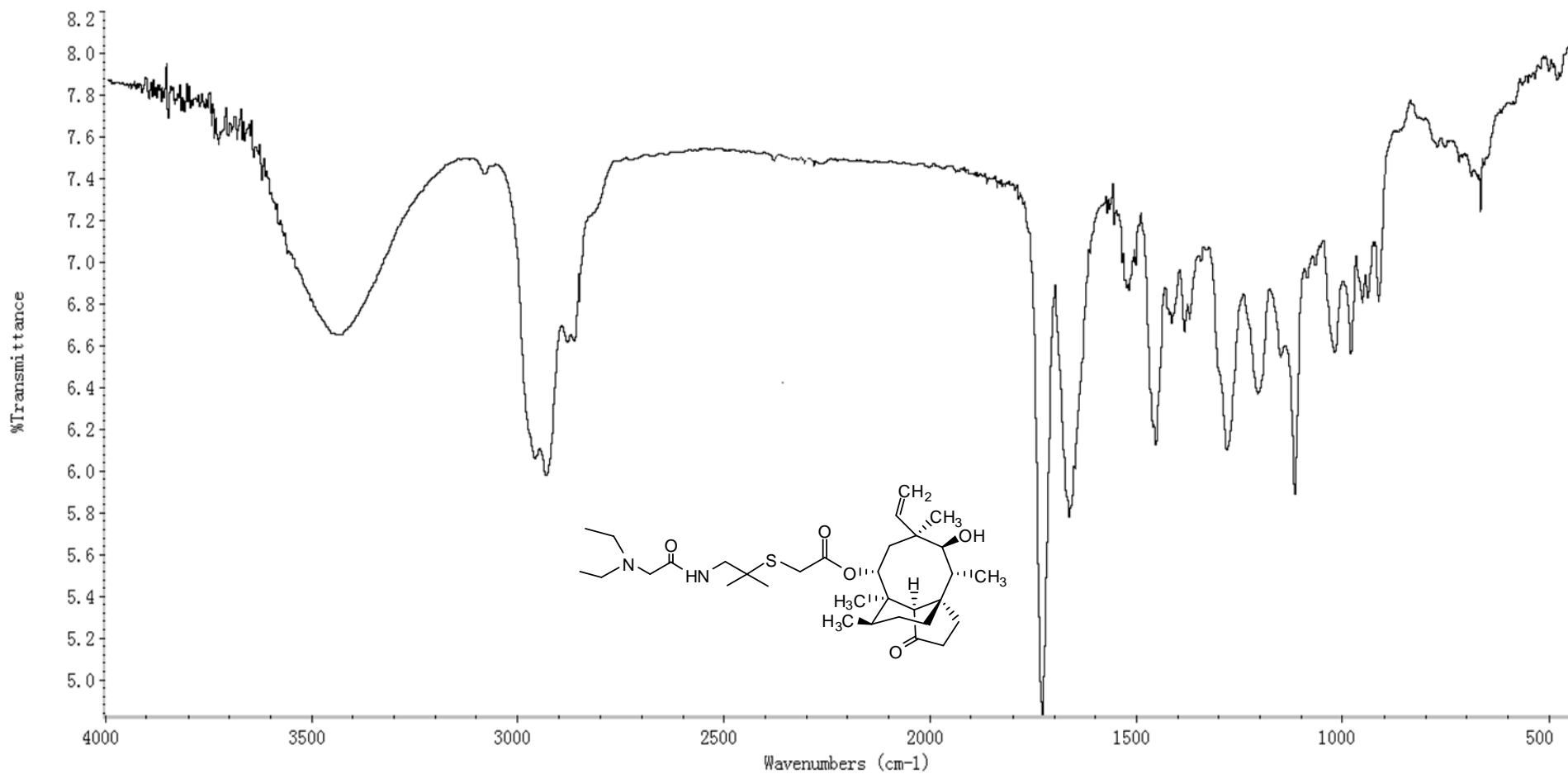

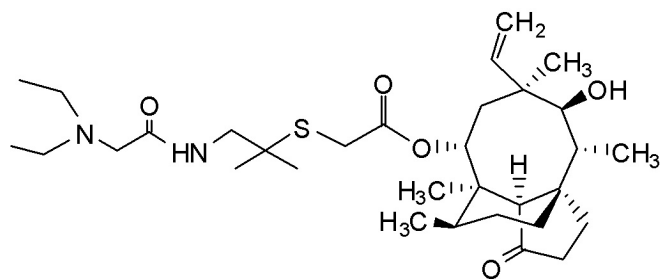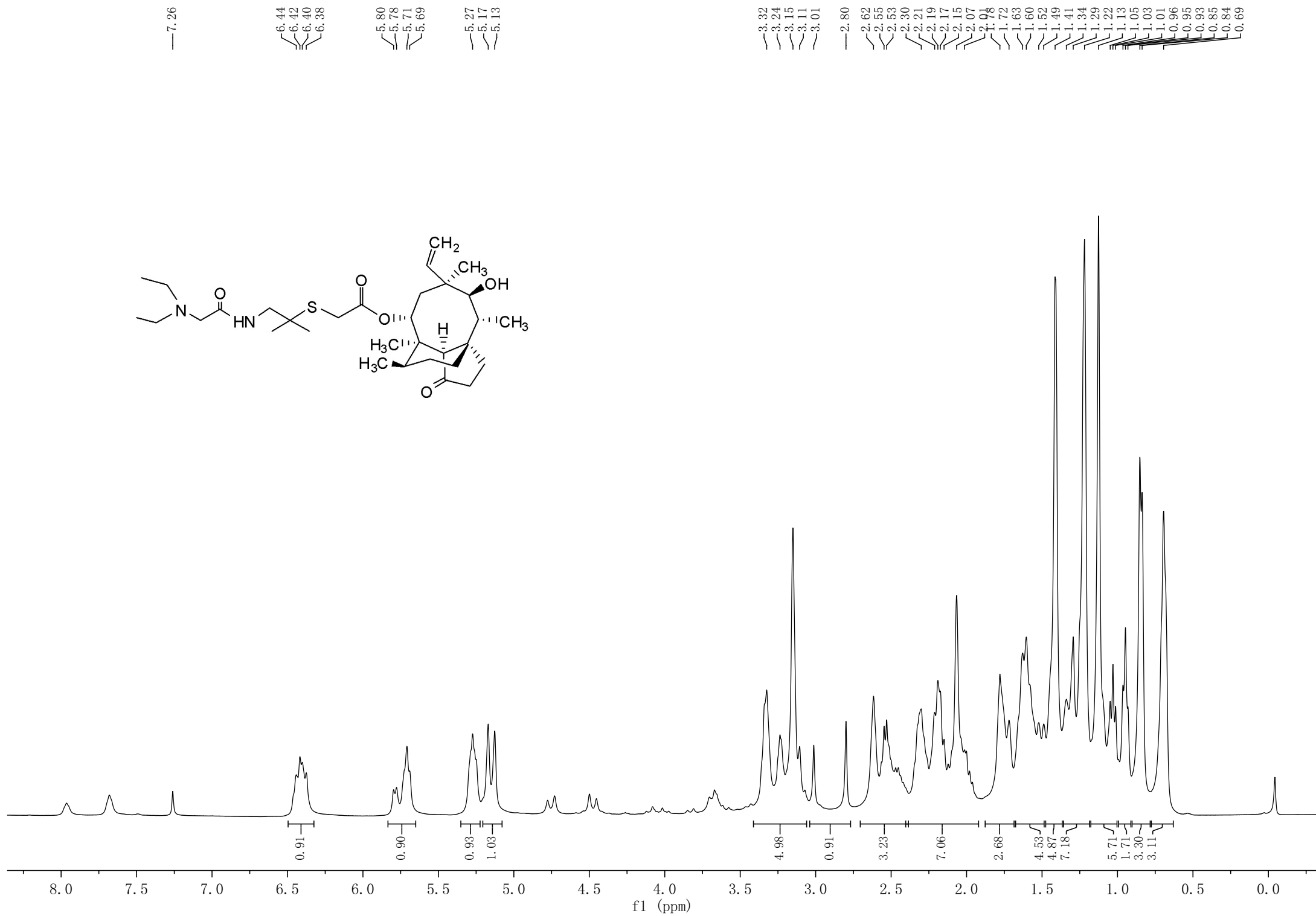

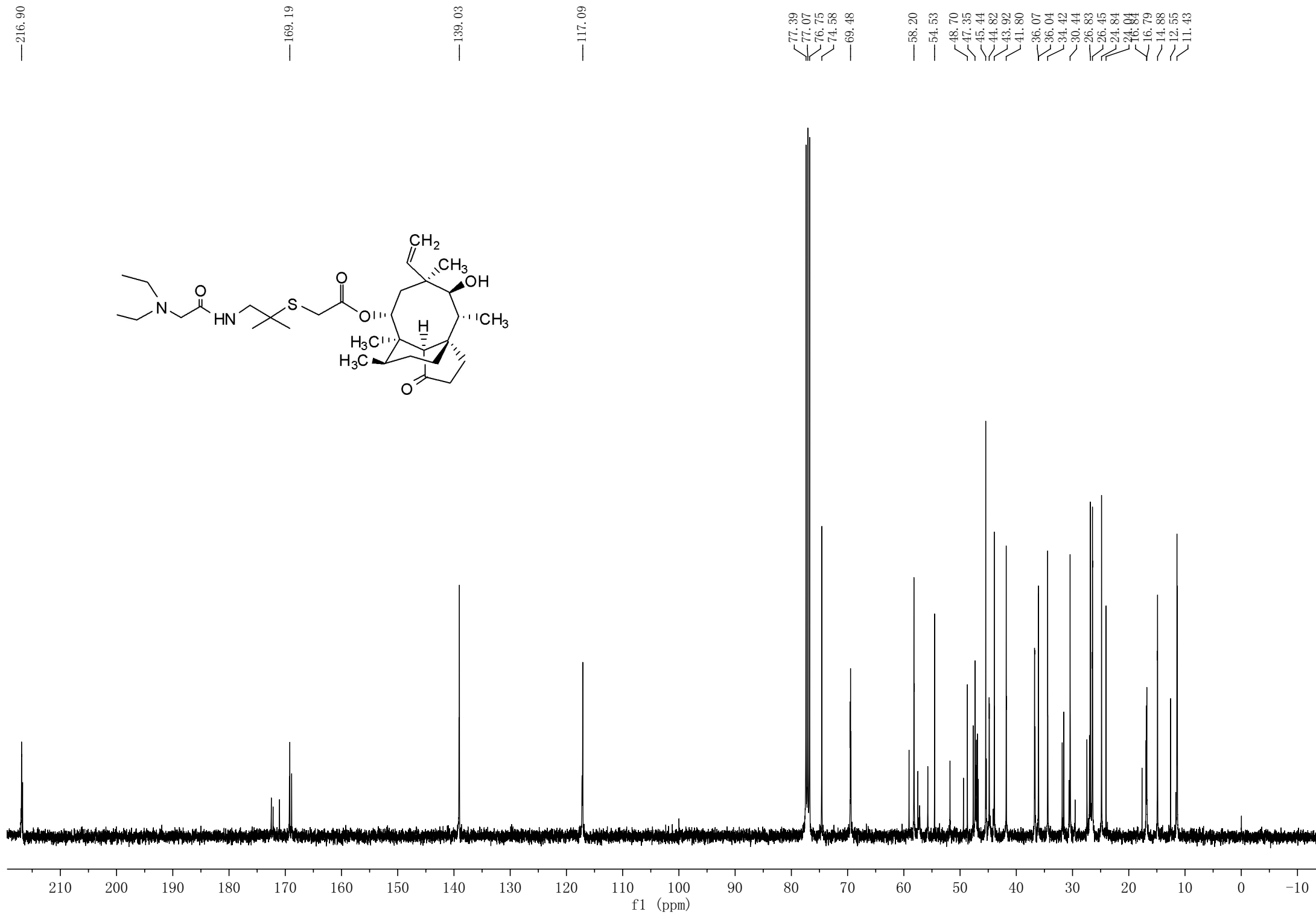

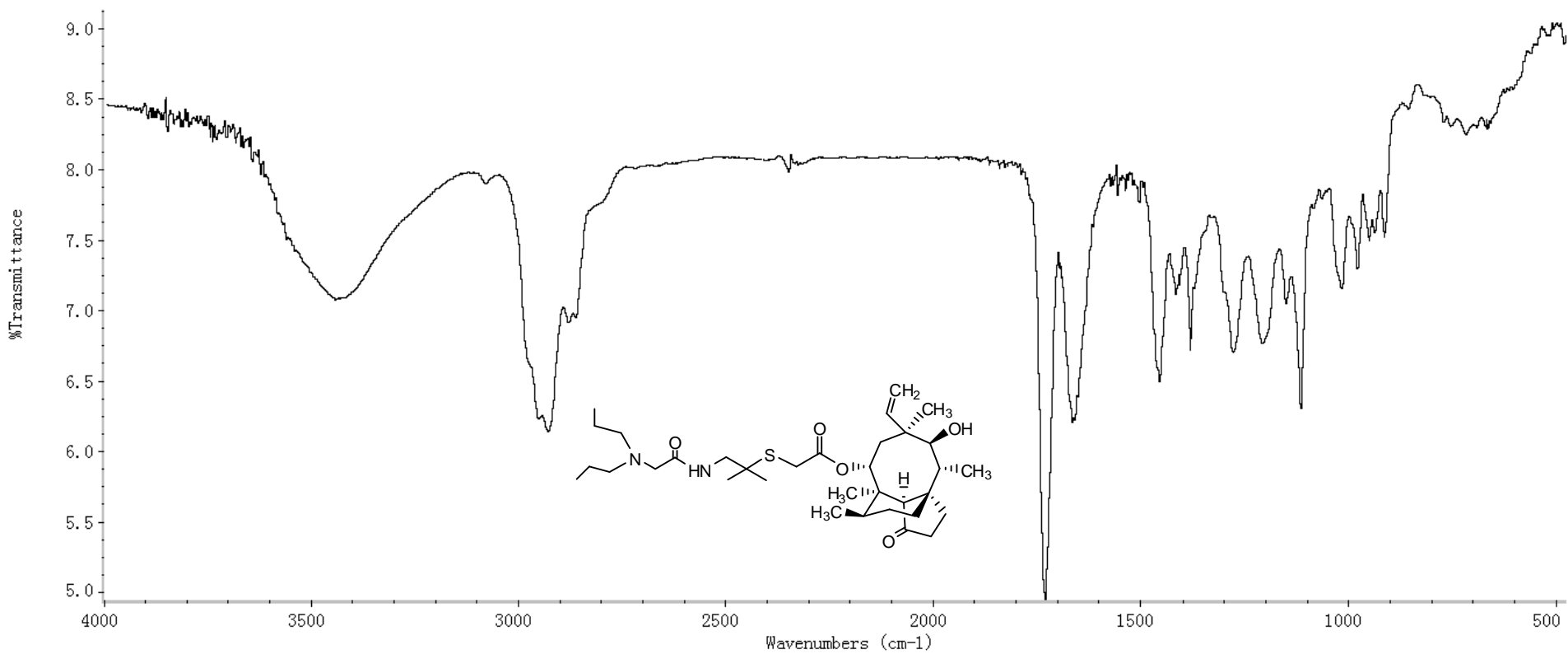

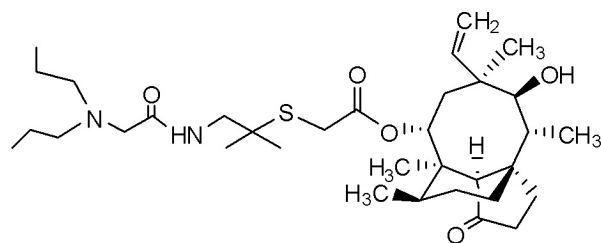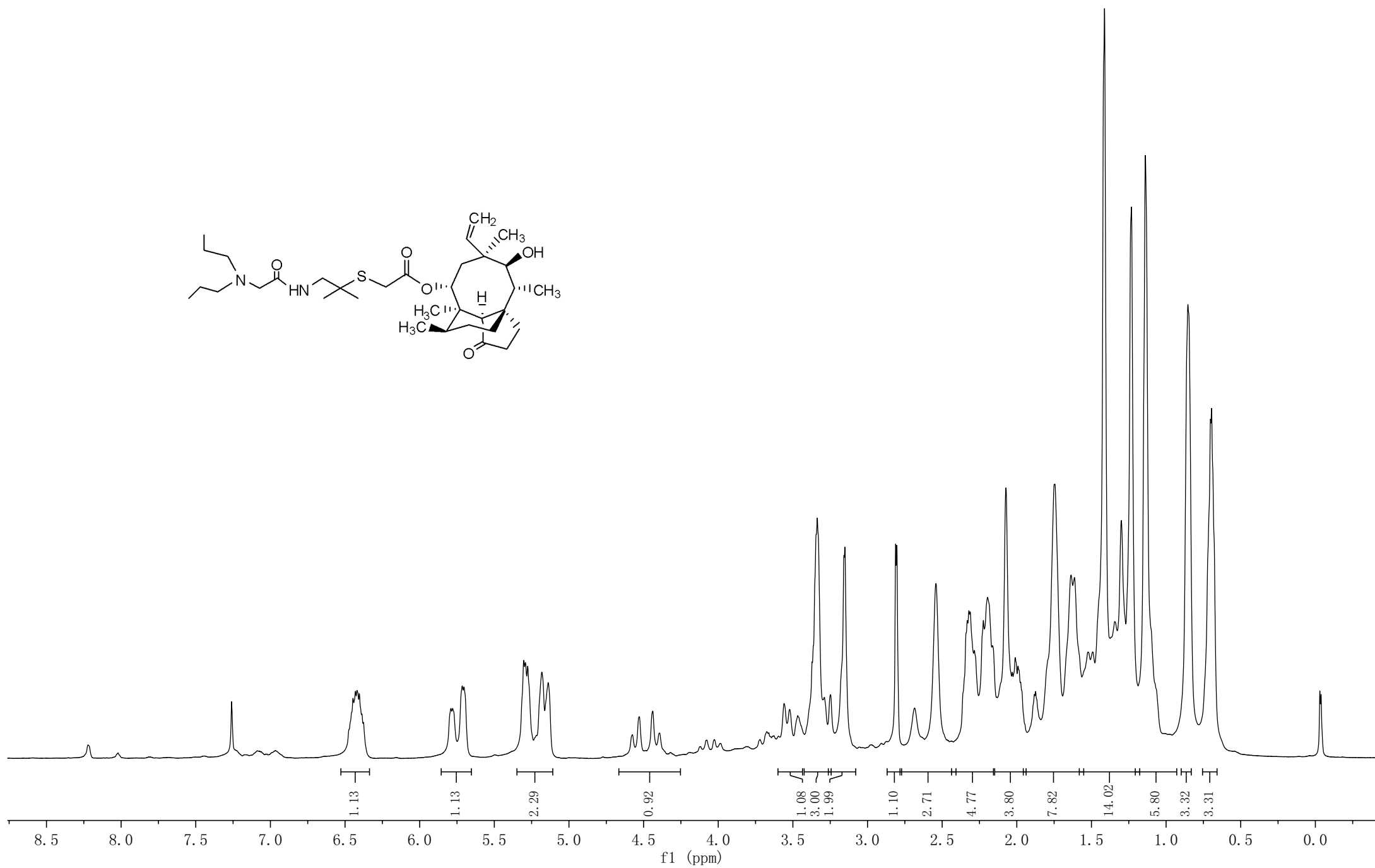

— 7.26

6.45  
6.43  
6.42  
6.40  
6.38

5.79  
5.78  
5.71  
5.70

5.30  
5.29  
5.28  
5.18  
5.14

4.57  
4.53  
4.44  
4.39

3.56  
3.52  
3.47  
3.37  
3.34  
3.29  
3.25  
3.16  
3.15

2.81  
2.80  
2.68  
2.54

2.33  
2.32  
2.31  
2.28  
2.22  
2.20  
2.16  
2.07  
1.74  
1.64  
1.61  
1.49  
1.41  
1.34  
1.30  
1.23  
0.85  
0.75  
0.70  
0.70

-0.03  
-0.04

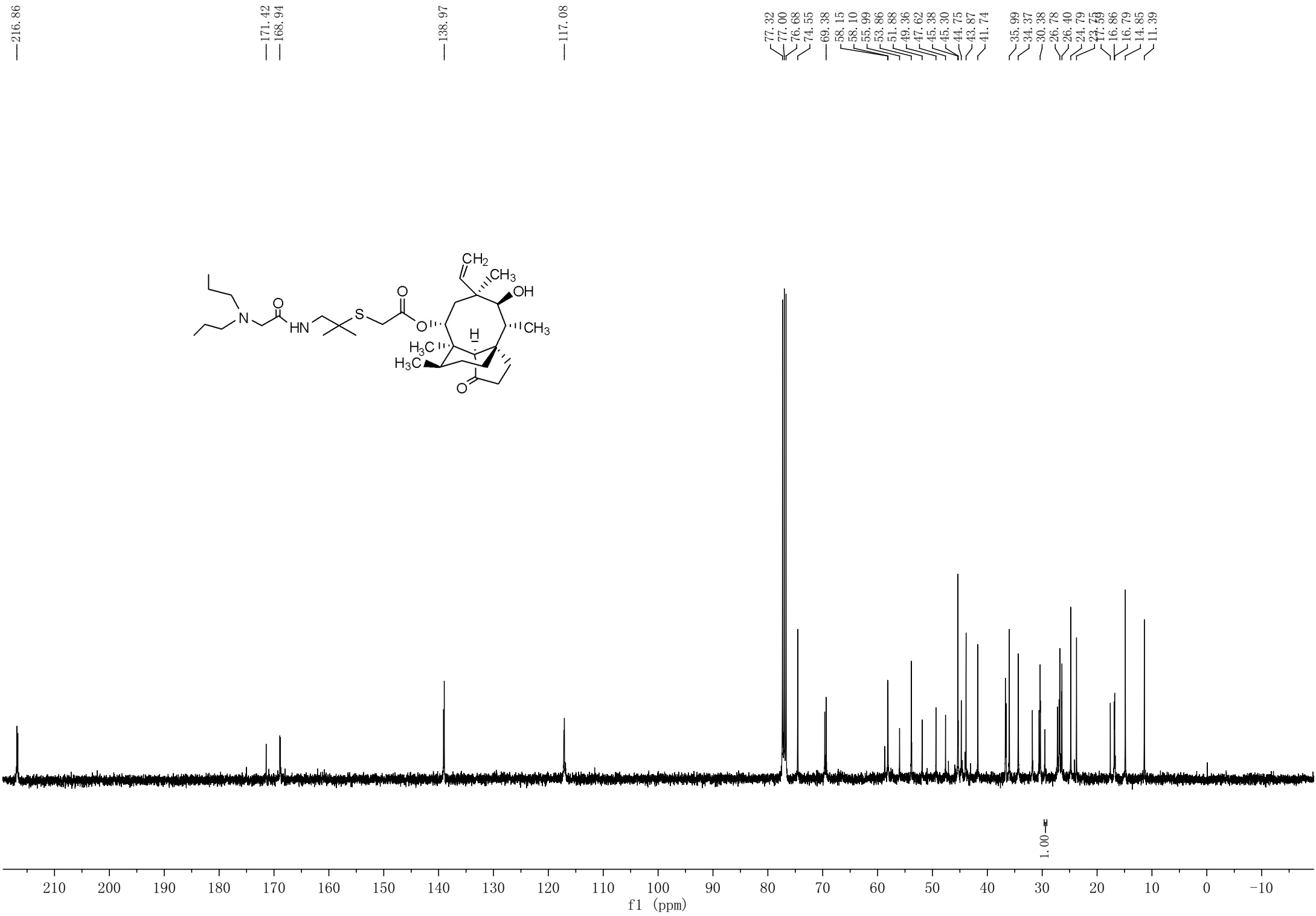

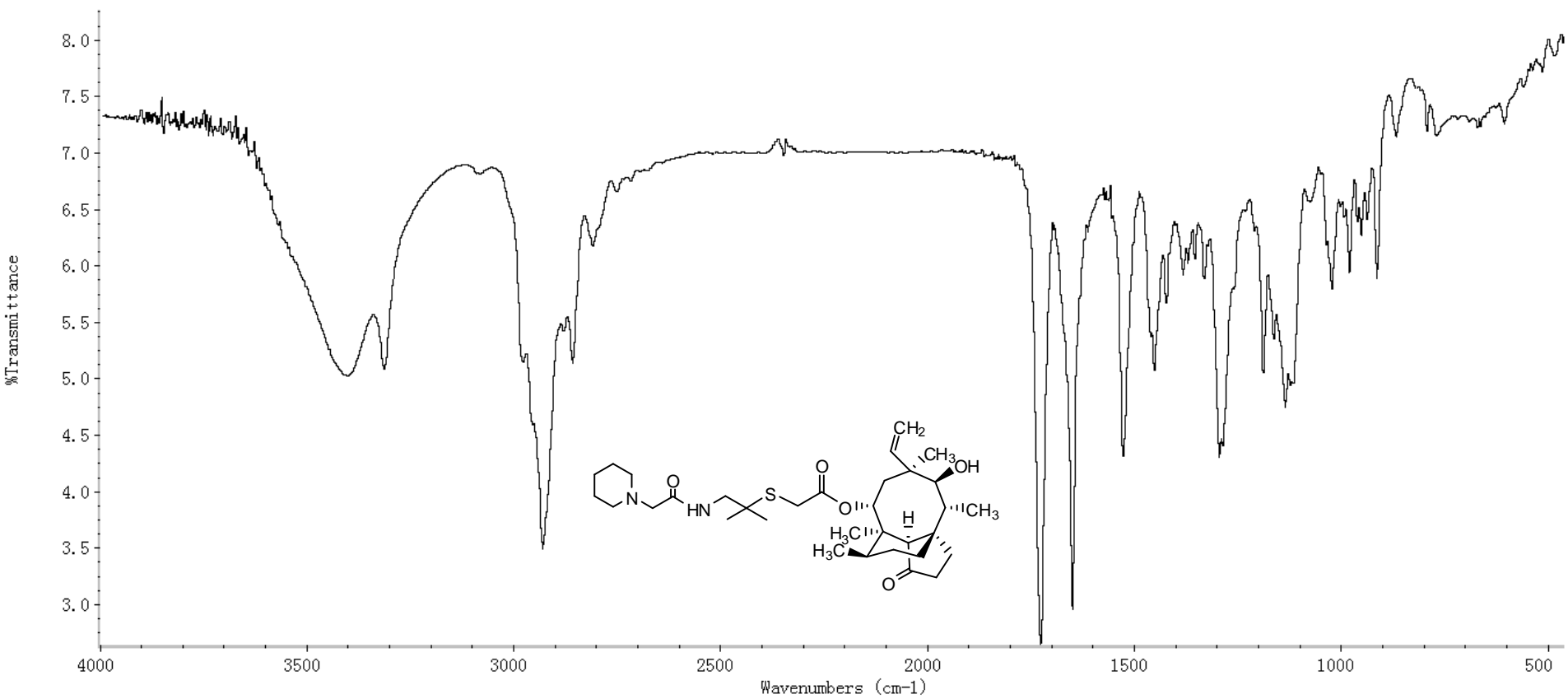

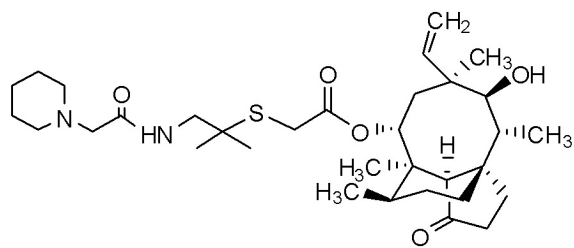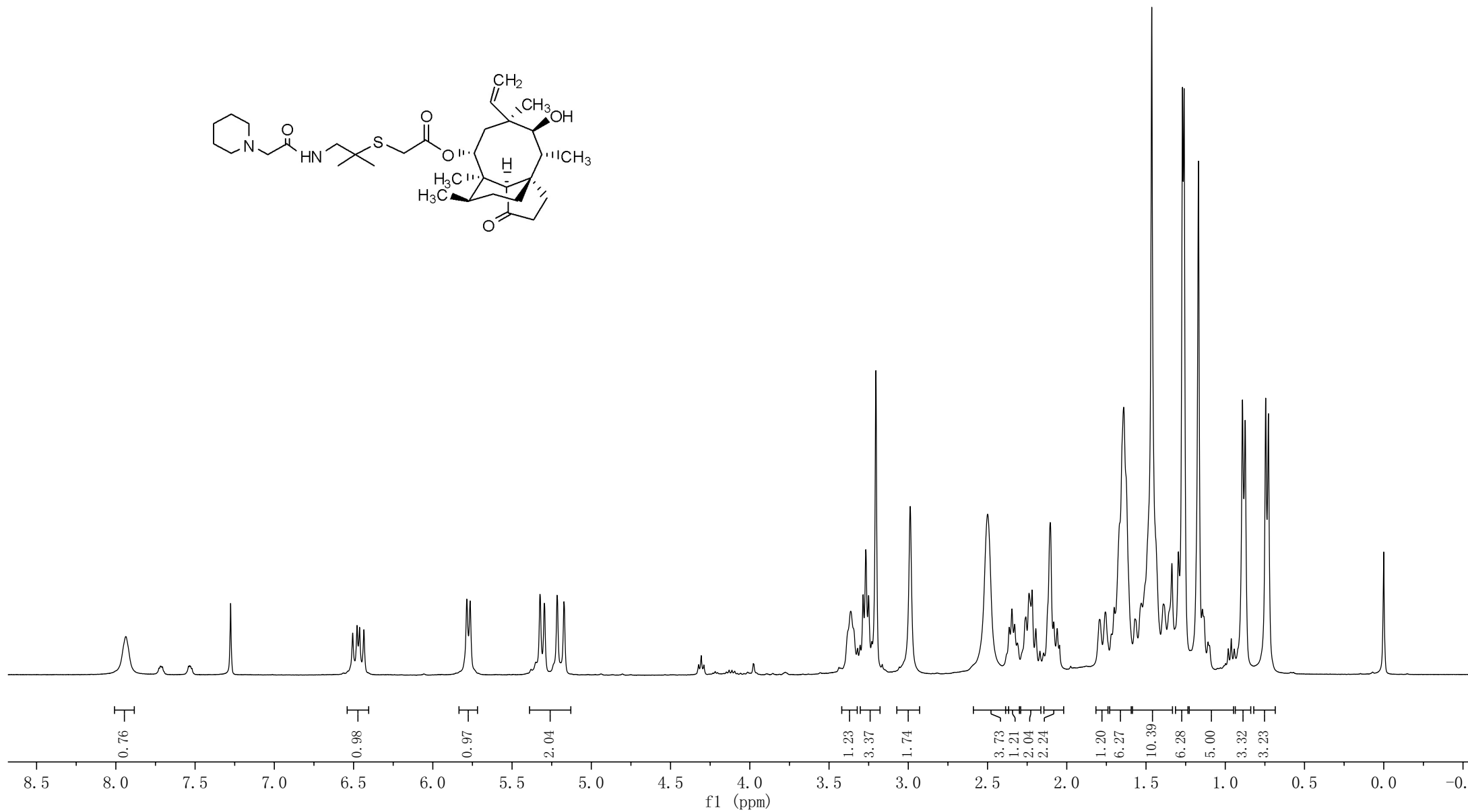

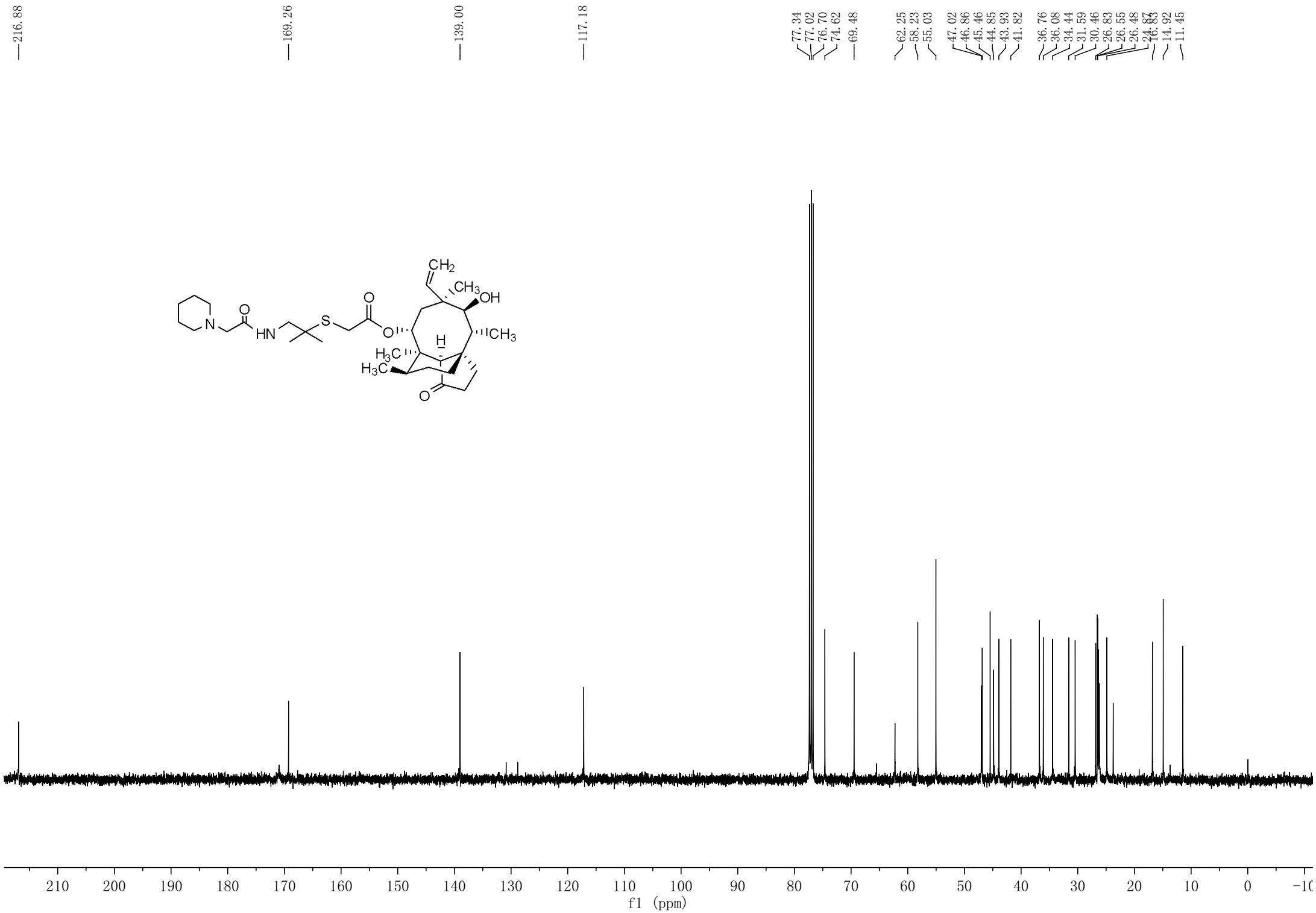

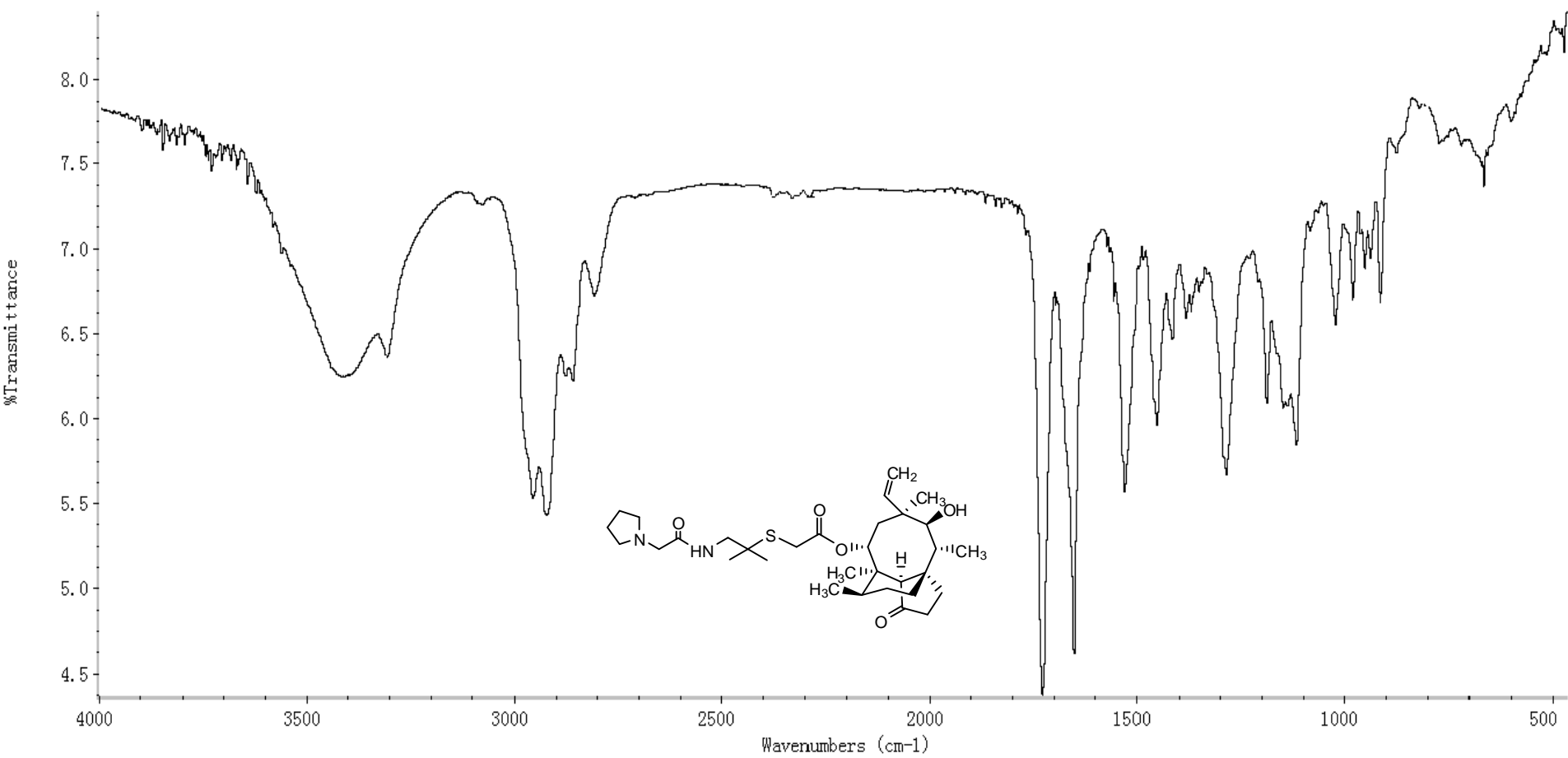

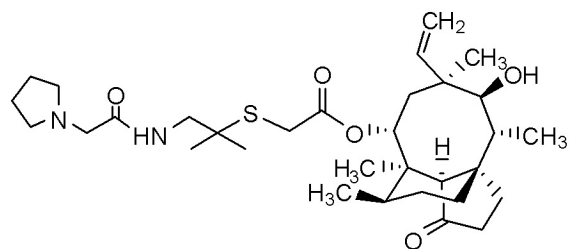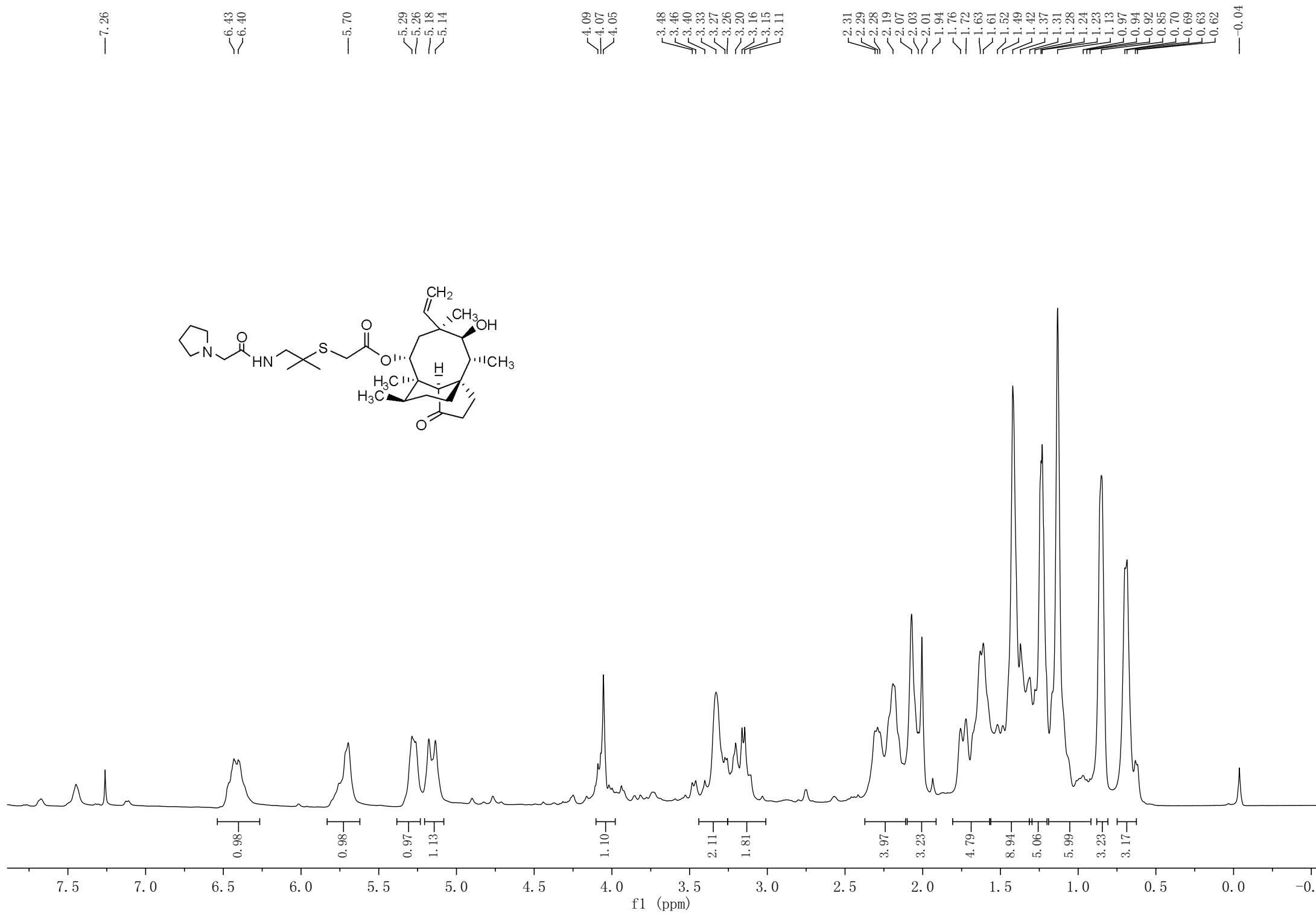

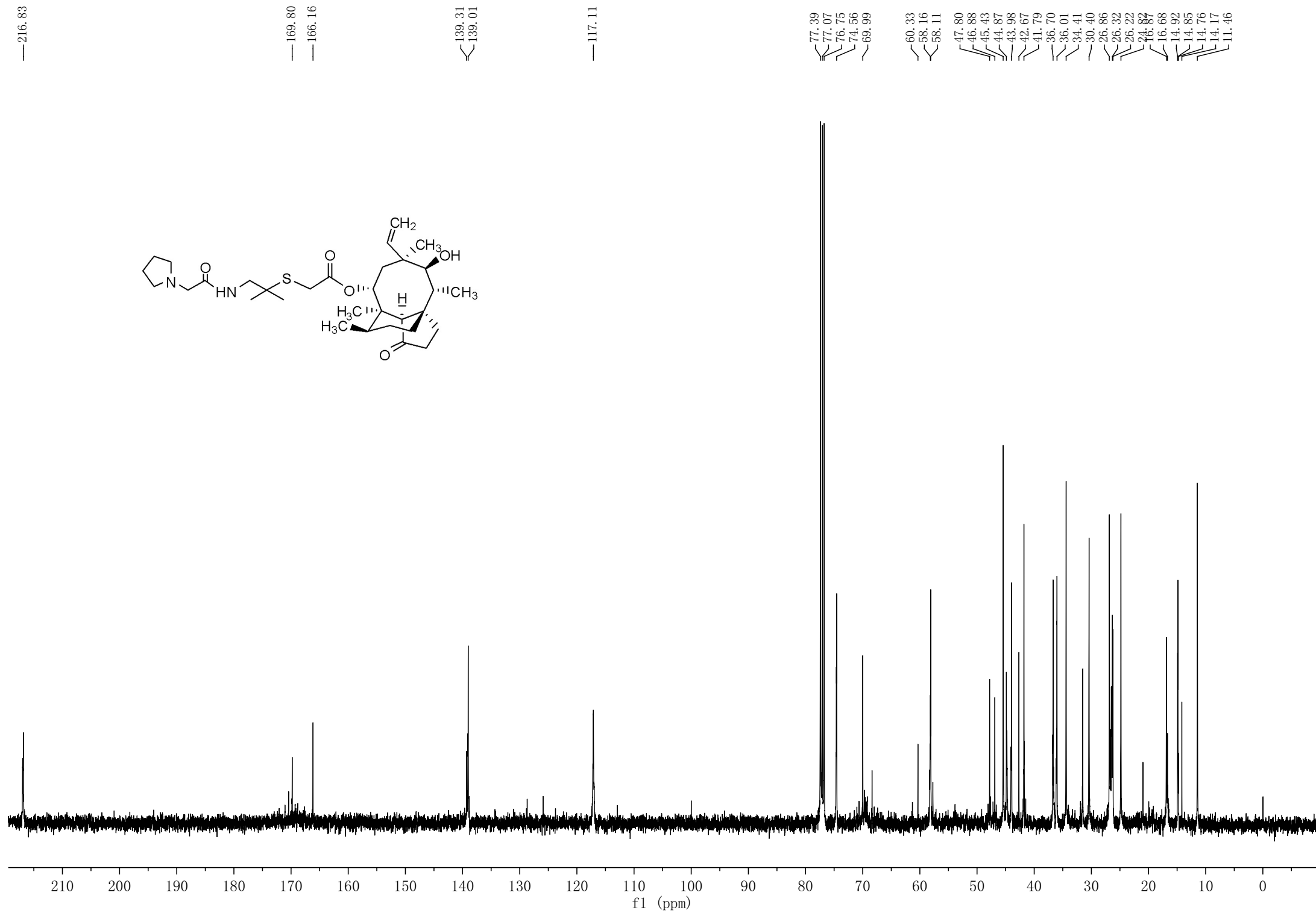

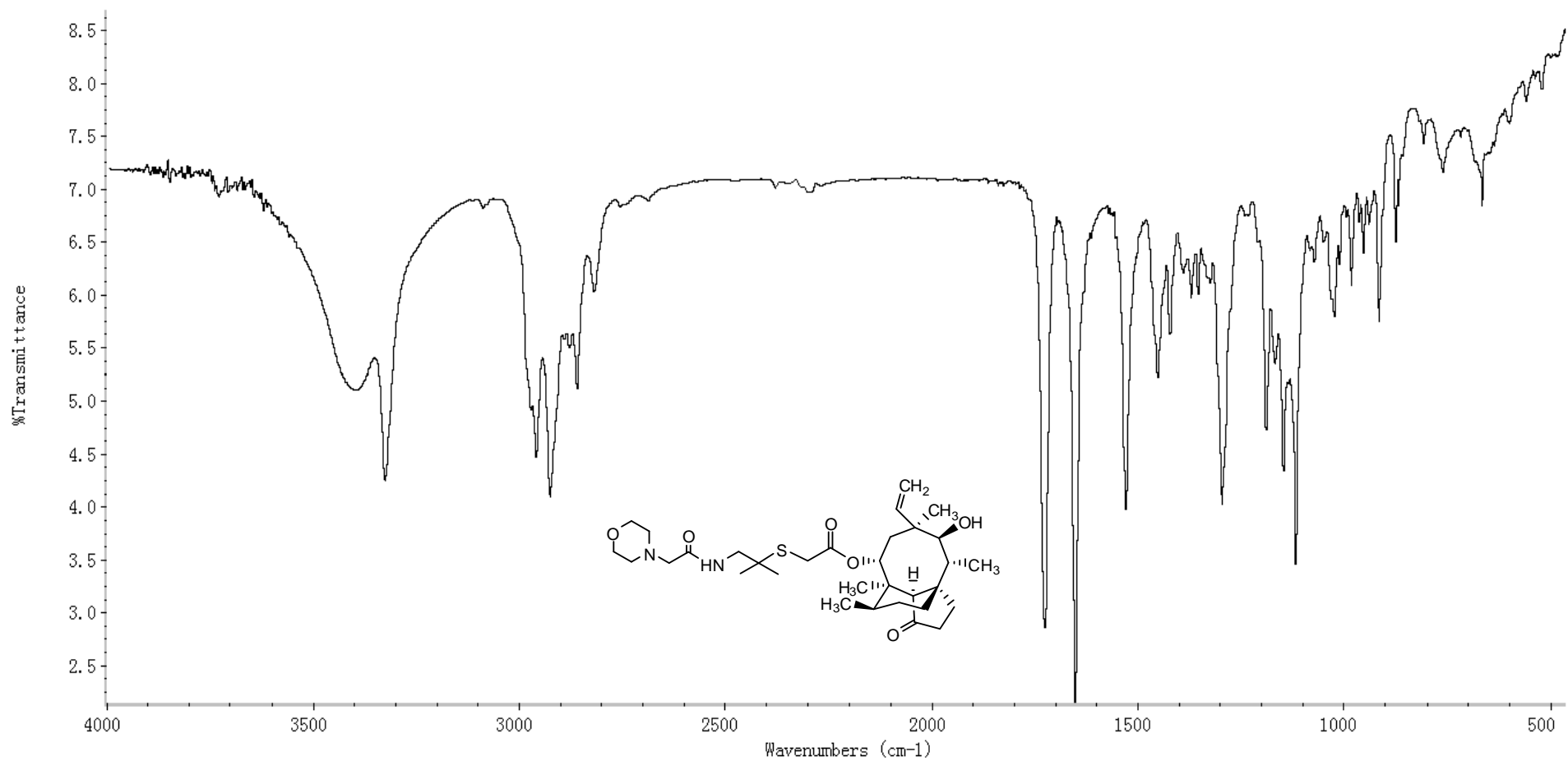

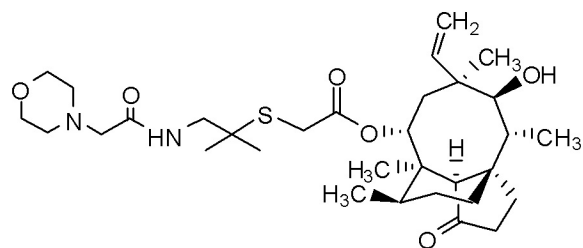

7.27

6.44  
6.41  
6.39  
6.36

5.74  
5.72

5.26  
5.23  
5.18  
5.13

3.75  
3.73  
3.72

3.34  
3.28  
3.26  
3.24  
3.23  
3.21  
3.19  
3.17  
3.16  
3.06  
2.55  
2.54

2.30  
2.20  
2.19  
2.18  
2.09  
2.07

1.63  
1.60  
1.45  
1.45  
1.42  
1.29  
1.25  
1.23  
1.21  
0.88  
0.85  
0.70  
0.68

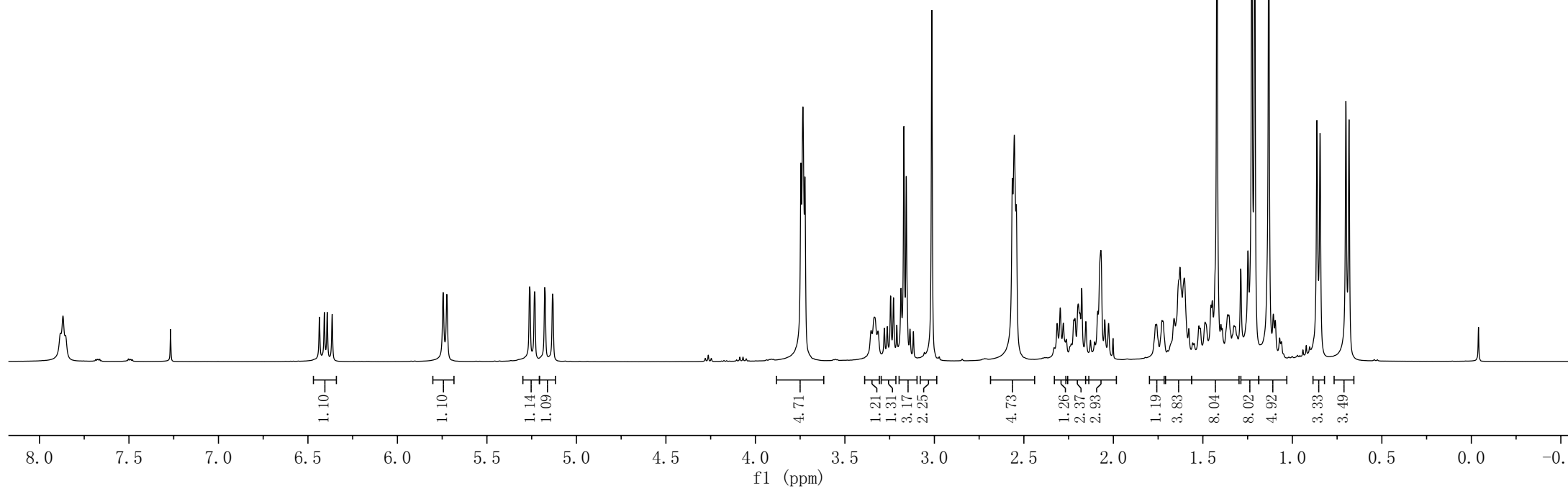

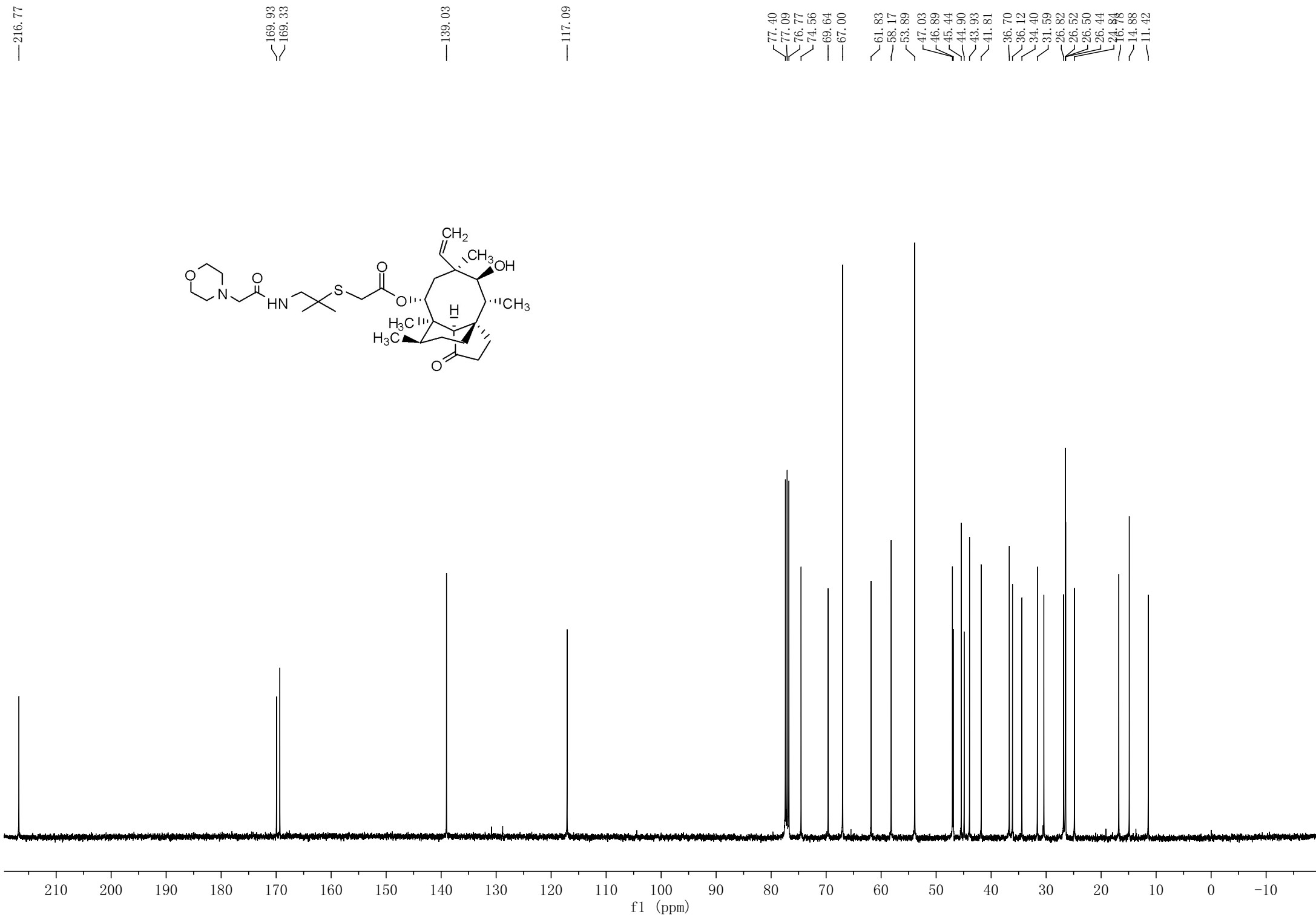

Supplement: Figure S1 — IR, 1H and13C NMR spectra of compounds 1–5f. (PDF) [file pone.0082595.s001.pdf]
