# Supplementary material for: Chemical Synthesis and Biological Activities of Novel Pleuromutilin Derivatives with Substituted Amino Moiety
Source: PLoS One. 2013 Dec 23;8(12):e82595. doi: 10.1371/journal.pone.0082595 (PMC3871055; doi:10.1371/journal.pone.0082595)
Supplement: Table S1 — Bind free energy, number of noncovalent molecular interaction and RMSD. (DOCX) [file pone.0082595.s002.docx]

**Table S1** Bind free energy, number of noncovalent molecular interaction and RMSD.

| Compound | ΔG_b_  (kcal/mol) | Noncovalent molecular interaction | | | | RMSD  (Å) |
| --- | --- | --- | --- | --- | --- | --- |
|  |  | hydro I interaction | Atom of Compound | Residue | Distance(Å) |  |
| 5a | -12.56 | H-bonding  H-bonding  H-bonding | OH (eight-membered ring)  C=O (ester)  NH (terminal) | G-2484  G-2044  C-2565 | 2.2  3.1, 3.2  2.1 | 0.96 |
| 5b | -13.42 | H-bonding  H-bonding  cation–π interaction | OH (eight-membered ring)  C=O (ester)  N (terminal) | G-2484  G-2044  A-2045 | 2.1  3.2, 3.0  4.2 | 1.20 |
| 5c | -12.23 | H-bonding  H-bonding | OH (eight-membered ring)  C=O (ester) | G-2484  G-2044 | 2.2  3.1, 3.4 | 0.94 |
| 5d | -13.16 | H-bonding  H-bonding | OH (eight-membered ring)  C=O (ester) | G-2484  G-2044 | 2.2  3.1, 3.2 | 1.07 |
| 5e | -12.90 | H-bonding  H-bonding | OH (eight-membered ring)  C=O (ester) | G-2484  G-2044 | 2.2  3.1, 3.2 | 1.15 |
| 5f | -12.35 | H-bonding  H-bonding | OH (eight-membered ring)  C=O (ester) | G-2484  G-2044 | 2.1  3.1, 3.1 | 1.21 |
