# Supplementary material for: Chemical Synthesis and Biological Activities of Novel Pleuromutilin Derivatives with Substituted Amino Moiety
Source: PLoS One. 2013 Dec 23;8(12):e82595. doi: 10.1371/journal.pone.0082595 (PMC3871055; doi:10.1371/journal.pone.0082595)
Supplement: Table S2 — Hydrogen bond lengths (Å) and bond angles (°) of compound 4. (DOCX) [file pone.0082595.s003.docx]

**Table S2** Hydrogen bond lengths (Å) and bond angles (°) of compound **4**

| D–H⋅⋅⋅A | d(D–H) | d(H⋅⋅⋅A) | d(D⋅⋅⋅A) | ∠DHA |
| --- | --- | --- | --- | --- |
| O(1) –H(1)⋅⋅⋅O(6)^a^  N(5) –H(5)⋅⋅⋅Cl(1)  N(5) –H(5)⋅⋅⋅O(4)  C(1) –H(1A)⋅⋅⋅O(4)  C(14)–H(14)⋅⋅⋅O(2)  C(17)–H(17B)⋅⋅⋅O(1)  C(21)–H(21B)⋅⋅⋅O(1)^b^  C(23)–H(23B)⋅⋅⋅O(6)  C(25)–H(25A)⋅⋅⋅O(2)^c^  C(28)–H(28A)⋅⋅⋅O(3)  C(28)–H(28B)⋅⋅⋅O(2) | 0.82  0.86  0.86  0.98  0.98  0.96  0.97  0.97  0.97  0.96  0.96 | 2.06  2.47  2.30  2.33  2.60  2.42  2.46  2.50  2.43  2.33  2.32 | 2.880(3)  2.950(3)  3.003(3)  2.738(3)  3.273(3)  2.753(4)  3.412(3)  2.839(3)  3.402(4)  2.730(3)  2.980(3) | 174  116  139  104  126  100  167  100  177  104  125 |

**Symmetry codes:** (a) -1+x, y, z; (b) -x, -1/2+y, 1-z; (c) -x, 1/2+y, -z.
