# Supplementary material for: Chemical Synthesis and Biological Activities of Novel Pleuromutilin Derivatives with Substituted Amino Moiety
Source: PLoS One. 2013 Dec 23;8(12):e82595. doi: 10.1371/journal.pone.0082595 (PMC3871055; doi:10.1371/journal.pone.0082595)
Supplement: Table S3 — Crystallographic Data for Intermediate 4. (DOCX) [file pone.0082595.s004.docx]

**Table S3**  Crystallographic Data for Intermediate **4**

| parameter | data | parameter | data |
| --- | --- | --- | --- |
| Empirical formula  Color, shape  Crystal size  Crystal system  Space geoup  *a* (Å)  *b* (Å)  *c* (Å)  *α* (º)  *β* (º)  *γ* (º)  V(Å)  Z  Temperature (K) | C_28_H_44_ClNO_5_S  clear light bronze, block  0.34×0.32x0.21mm  Monoclinic  P1211 (no. 4)  13.0600(4)  8.7981(2)  13.2290(4)  90  110.453(3)  90  1424.22(7)  2  293(2)K | θ range for data collection  Wavelength  Radiation type  Measurement method  *R*_int_  Refinement method  Index ranges(*h, k, l*)  Independent reflections  Threshold expression  R_gt_ (F)  wR_ref_ (F^2^)  S | 3.9060~27.4560º  0.7107  Mo K\a  ω scan  0.0157  Full-matrix; F^2^  -16 ≤ h ≤ 16,  -9 ≤ *k* ≤ 10,  --15 ≤ *l* ≤ 16  4839  *I* >2*σ*(*I*)  0.0373  0.0869  1.039 |
